# Large-scale societal dynamics are reflected in human mood and brain

**DOI:** 10.1038/s41598-022-08569-3

**Published:** 2022-03-17

**Authors:** Alexander V. Lebedev, Christoph Abé, Kasim Acar, Gustavo Deco, Morten L. Kringelbach, Martin Ingvar, Predrag Petrovic

**Affiliations:** 1 Department of Clinical Neuroscience, Karolinska Institutet, Stockholm, Sweden; 2 Center for Cognitive and Computational Neurosceince (CCNP), Karolinska Institutet, Stockholm, Sweden; 3 Center for Brain and Cognition, Computational Neuroscience Group, Department of Information and Communication Technologies, Universitat Pompeu Fabra, Barcelona, Spain; 4 Institució Catalana de la Recerca i Estudis Avançats (ICREA), Barcelona, Spain; 5 Centre for Eudaimonia and Human Flourishing, Linacre College, University of Oxford, Oxford, UK; 6 Department of Psychiatry, University of Oxford, Oxford, UK; 7 Center for Music in the Brain, Aarhus University, Aarhus, Denmark

**Keywords:** Human behaviour, Environmental economics, Criticality, Epidemiology, Reward, Social behaviour, Stress and resilience, Amygdala, Insula, Limbic system, Prefrontal cortex

## Abstract

The stock market is a bellwether of socio-economic changes that may directly affect individual well-being. Using large-scale UK-biobank data generated over 14 years, we applied specification curve analysis to rigorously identify significant associations between the local stock market index (FTSE100) and 479,791 UK residents’ mood, as well as their alcohol intake and blood pressure adjusting the results for a large number of potential confounders, including age, sex, linear and non-linear effects of time, research site, other stock market indexes. Furthermore, we found similar associations between FTSE100 and volumetric measures of affective brain regions in a subsample (n = 39,755; measurements performed over 5.5 years), which were particularly strong around phase transitions characterized by maximum volatility in the market. The main findings did not depend on applied effect-size estimation criteria (linear methods or mutual information criterion) and were replicated in two independent US-based studies (Parkinson’s Progression Markers Initiative; n = 424; performed over 2.5 years and MyConnectome; n = 1; 81 measurements over 1.5 years). Our results suggest that phase transitions in the society, indexed by stock market, exhibit close relationships with human mood, health and the affective brain from an individual to population level.

## Introduction

Stock markets mirror the underlying socio-economic status of a population^1,2^ and may therefore be used as an index or bellwether of the global societal dynamics. Previous research has suggested that capital market evolution exhibits strong impact on traders' emotional states^3^, but is also associated with the welfare of individuals who have no direct involvement in the stock market^4^. More specifically, it has been suggested that stock market turbulence is linked to increased anxiety^5^, self-harm and suicide rates^6–8^, elevated levels of binge drinking^9^ and fatal car accidents^9,10^ in the society. These effects may be particularly pronounced in long-lasting socioeconomic events, such as the 2008 stock market crash or the economy slowing in the COVID19 pandemic.

To date, there are no studies that have investigated the association of market behaviour with brain function and structure. In a broader perspective, previous research has suggested that the events that happen in the society have a clear impact on the brain. For example, previous studies have identified reductions in the prefrontal cortex following earthquakes^11^ and warzone experiences^12^. One study has demonstrated how a single extreme aversive global event may impact fear circuits by linking individuals’ geographical proximity to the site of 9/11 terrorist attacks to the reactivation of the amygdala during memory recollection^13^. In line with this, a recent study has suggested that intense experience of the COVID19 outbreak is linked to volumetric increase of the amygdala^14^.

The present study aims to understand whether more subtle but frequently occurring global events may leave a trace in the human brain on a population level. Here, we investigated how fluctuations in the stock market are associated with brain structure. Since such fluctuations also mirror global socioeconomic changes in the society^1^, the investigated associations imply a broader perspective than the specific effects of the market per se.

## Methods

To address the main research question, we accessed structural MRI data of 39,755 UK citizens from the UK Biobank acquired over approximately 5.5 years (between 2014-05-02 and 2019-10-31). Initial enrolment of the UK Biobank study took place over four years starting from 2006. It targeted UK citizens aged between 40 and 69 with demographics distribution at recruitment corresponding to such of Great Britain as a whole at the 2001 Census with a plan to follow them for at least 30 years thereafter. No explicit or implicit inclusion criteria were applied that would make the study sample non-representative of the UK population with respect to stock market participation.

Once the data transfer had been completed, we matched the scan date with the corresponding information on the Financial Times Stock Exchange 100 Index (FTSE100) characterizing stock price of the top 100 UK companies with the largest revenue, which was our main independent variable (see Supplementary Fig. S1 and Supplementary Table S1 for description of the whole dataset, which also included mood data collected over a period of approximately 14 years). The FTSE100 was chosen because the study subjects resided in the UK, and local changes in the economy were expected to impact brain structure on a population level most strongly. Capital market indexes were prioritized over other socioeconomic indicators due to superior time resolution, more extensive previous research and ease of use with respect to acquiring standardized data for multiple countries. It is worth noting, however, that we have also replicated our main findings with alternative metrics (housing prices, unemployment rates, see Supplementary S13–15) and controlled for them in a separate set of analyses (Supplementary S16). In the investigated period, FTSE100 exhibited negative association with unemployment rates (Supplementary S1, S13) and positive with housing prices (Supplementary S13).

In order to index effects on the brain, daily time-series of the market capital index was matched with neuroimaging data focusing on a set of preregistered (https://osf.io/h52gk) brain regions known to play key roles in the processing of rewards and losses, as well as threat and fear^15–18^: amygdala, nucleus accumbens, insula, anterior, subcallosal and dorsal cingulate and lateral orbitofrontal cortical areas. Abnormal functioning of these circuits has also been documented to play a key role in the pathophysiology of anxiety and depression^19–22^. The total sample size was deemed appropriate for studying the afore-mentioned associations, and met the determined minimum of 20,000 data-points to test significance of a small effect-size (r = 0.04) with statistical power of > 0.9 determined in G*Power, version 3. Previous research suggests that brain morphometry is capable of capturing plastic changes that happen after weeks^23^ or days^24^ of engagement of the relevant brain networks. Moreover, even acute activation of brain networks is associated with noticeable alterations in morphometric measures^25^. Even though these changes may represent widely different underlying mechanisms depending on observational time-scales, the literature supports the idea that grey matter changes in major brain networks occur in parallel with their functional reorganization^26^.

Prior to the main analysis, we attempted to replicate previous behavioural findings suggesting a relation of market fluctuations with mood and well-being^4,7,27,28^ on a large sample from the UK Biobank data (n = 479,791) collected over a period of approximately 14 years.

We employed mixed-effect linear modelling to investigate the studied associations and used the Chinese capital market index as a reference, adhering to the preregistered workflow. However, the protocol was further amended to implement more comprehensive adjustment for nuisance covariates, including research site, age, sex, linear and non-linear effects of time, psychiatric diagnosis, reference indexes, intracranial volume, as well as all possible combinations of the selected confounds in the specification curve analyses^29^. The specificity of the investigated associations was assessed in a series of equivalent analyses of the capital stock market indexes of the UK’s 15 top trading partners, sociocultural distances of the UK from 17 countries leveraging data from Liu et al., 2018^30^ and other global candidate metrics with 1/f properties (UK seismic activity and mortality rates). The main results were also replicated for different time-bins and frequency bands, tested against several types of noise generated under empirical null assumptions, validated employing generalized additive modelling^31^ and mutual information criterion to address limitations of linear methods^32^. Even considering all precautions taken, it is important to recognize that the UK Biobank study protocol did not employ explicit stratification for participants’ socioeconomic status, which may bias sampling procedure due to its possible sensitivity to global socioeconomic environment.

Whenever possible, the statistical results were supplemented with effect-sizes for different time scales. We also performed exploratory temporal causality analysis employing Toda-Yamamoto tests specifically designed for serially correlated data^33^.

A number of external validation analyses have been conducted. Specifically, we attempted to replicate imaging and non-imaging results in two independent United States datasets: (1) Parkinson’s Progression Markers Initiative (PPMI, www.ppmi-info.org), n = 424, performed over 2.5 years and (2) MyConnectome (http://myconnectome.org), n = 1, 81 measurements over 1.5 years.

## Results

**Figure 1:**
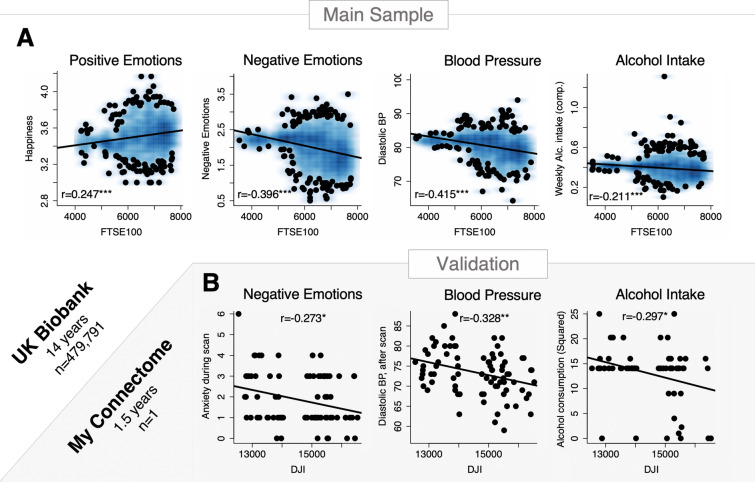
Non-MRI variables and stock market moves. The figure illustrates the identified associations between stock market moves and non-MRI indicators of well-being in the UK Biobank sample (top panel **A**) and My Connectome data, a single-subject study (bottom panel **B**); *p < 0.05, **p < 0.01, ***p < 0.001. Corresponding effect-sizes estimated with mutual information criterion are reported in the supplement (Supplementary Table S11).

Analysing the relations between FTSE100 and self-reported measures of emotional well-being we confirmed that market ups (higher FTSE100 scores) were associated with higher scores of “happiness” and lower scores in self-reported “negative emotional facets”: irritability, hurt and nervous feelings, anxiety (Fig. 1; Table 1). The identified association also held true for the 5.5-years of the MRI subsample (Supplementary Table S2). We further explored non-imaging variables that are associated with mood changes, i.e. alcohol intake (overall intake frequency and a composite score reflecting weekly intake of all alcoholic beverages) and diastolic blood pressure (automatic readings in mmHg measured at rest), and showed that they were also highly correlated with the FTSE100 (Fig. 1A) in that both measures increased when the stock market decreased in value. Several of these effects (relation between stock market and negative emotions, blood pressure or alcohol-intake) were reproduced in the My Connectome data-set consisting of one single subject whose measurements were taken at 81 timepoints during a period or 1.5 years (Fig. 1B).

**Table 1:** Subjective well-being and FTSE100 scores: 14 years period. *β*_*std*_ standardized β coefficients, *p*_*fdr*_ p-values corrected for multiple testing with false discovery rate. Subcomponents of negative emotions are binary variables (–), *Day/MonthAVG* data averaged by days and months. The analyses leveraged random linear mixed effects framework with subject as a random effect, as a subset (n = 1427) of the study subjects was assessed twice. *Corresponding effect-sizes estimated with mutual information criterion are reported in the supplement (Supplementary Table S11).

	Linear mixed-effects			Effect-sizes*, Pearson r (95% CI)		
	*β*_*std*_	*T *(*df*)	*p*_*fdr*_	Raw	DayAVG	MonthAVG
NegEm (total)	− 0.03	− 24.33 (37,671)	< 0.001	− 0.034 (− 0.037, − 0.031)	− 0.396 (− 0.428, − 0.362)	− 0.591 (− 0.692, − 0.467)
Irritability	− 0.01	− 5.86 (37,671)	< 0.001	–	− 0.117 (− 0.155,− 0.078)	− 0.266 (− 0.418,− 0.099)
Sensitivity/hurt	− 0.04	− 24.75(37,671)	< 0.001	–	− 0.379 (− 0.412,− 0.345)	− 0.516 (− 0.632,− 0.378)
Nervous	− 0.01	− 8.51 (37,671)	< 0.001	–	− 0.264 (− 0.300,− 0.228)	− 0.508 (− 0.625,− 0.368)
Worrier/anxious	− 0.02	− 10.9 (37,671)	< 0.001	–	− 0.287 (− 0.322,− 0.25)	− 0.444 (− 0.572,− 0.294)
Happiness	0.04	19.81 (15,633)	< 0.001	0.052 (0.047,0.056)	0.247 (0.204,0.288)	0.556 (0.406,0.677)

**Figure 2:**
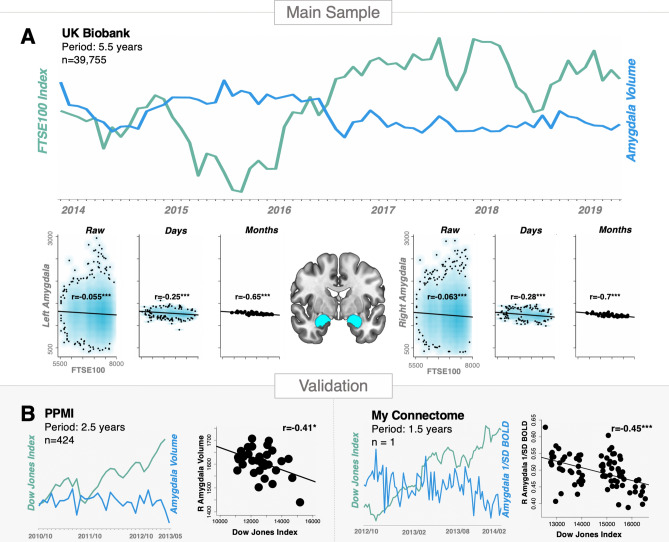
Studied brain-market associations. The figure illustrates the study rationale and reports the investigated effects for the main sample (**A**), as well as their replication (**B**) in a medium-sized (PPMI) and single-subject (My Connectome) fMRI study; *p < 0.05, ***p < 0.001. Raw-individual measures without day-averaging.

We then tested and confirmed our main hypothesis by showing that FTSE100 oscillations exhibited significant associations with the morphometry of the affective brain circuits. The most notable result was that bilateral amygdala, involved in threat detection and anxiety processing^18–22^, showed a negative relation with the UK economic performance (Fig. 2A, and Table 2, whole-brain analysis revealing that the effects are not limited only by the preregistered regions is reported in Supplementary Fig. S2). Similar (but in expectedly reversed direction) associations were found for alternative socioeconomic metrics (housing prices and unemployment rates, Supplementary Table S14). Of note, our results were replicated in an independent set of 424 individuals from the PPMI database, an clinical study targeting the US population (www.ppmi-info.org), and conceptually also in “My Connectome” single-subject longitudinal study^34^. In “My Connectome”, structural data was not publicly available, however, using BOLD-signal variability^35^ in the amygdala as a proxy biological measure demonstrated that our results also generalise to functional characteristics of the fear network (Fig. 2B). It is worth noting, however, that unlike the main results, detrending the Dow Jones index in these two (PPMI and MyConnectome) datasets reduced effect-sizes without reversing the direction of the associations (Supplementary Fig. S13).

**Table 2:** Associations between FTSE100 and structural characteristics of the fear network: cortical and subcortical volumes. *Day/MonthAVG* data averaged over days and months. Intracranial volume (ICV) was selected as a reference measure, which was not expected to exhibit significant associations with global stock market behaviour. *β*_*std*_ standardized β coefficients, *p*_*fdr*_ p-values corrected for multiple testing with false discovery rate. The analyses leveraged random linear mixed effects framework with subject as a random effect, as a subset (n = 1427) of the study subjects was scanned twice. *corresponding effect sizes estimated with the mutual information criterion are reported in the supplement (Supplementary Table S11).

Region	Linear mixed-effects			Effect-sizes*, Pearson r (95% CI)		
	*β*_*std*_	*T*_883_	*p*_*fdr*_	Raw, *n* = 30,775	DayAVG, *n* = 1299	MonthAVG, *n* = 66
L amygdala	− 0.054	− 9.51	< 0.001	− 0.055 (− 0.066, − 0.043)	− 0.253 (− 0.304, − 0.202)	− 0.615 (− 0.746, − 0.439)
R amygdala	− 0.062	− 10.91	< 0.001	− 0.063 (− 0.074, − 0.052)	− 0.282 (− 0.332, − 0.231)	− 0.644 (− 0.767, − 0.477)
L accumbens	− 0.054	− 9.54	< 0.001	− 0.055 (− 0.066 ,− 0.044)	− 0.232 (− 0.283, − 0.18)	− 0.623 (− 0.752, − 0.449)
R accumbens	− 0.062	− 10.89	< 0.001	− 0.064 (− 0.075, − 0.052)	− 0.259 (− 0.309, − 0.207)	− 0.662 (− 0.779, − 0.5)
L LOFC	− 0.026	− 4.68	< 0.001	− 0.031 (− 0.042, − 0.02)	− 0.141 (− 0.193, − 0.087)	− 0.443 (− 0.619, − 0.225)
R LOFC	− 0.019	− 3.49	0.001	− 0.023 (− 0.034, − 0.012)	− 0.082 (− 0.136, − 0.028)	− 0.292 (− 0.499, − 0.054)
L insula	0.037	6.62	< 0.001	0.042 (0.031, 0.053)	0.21 (0.157, 0.261)	0.494 (0.286, 0.657)
R insula	0.032	5.86	< 0.001	0.037 (0.026, 0.048)	0.187 (0.134, 0.239)	0.413 (0.19, 0.595)
L subcallosal	0.018	3.18	0.002	0.02 (0.009, 0.032)	0.134 (0.08, 0.187)	0.322 (0.087, 0.524)
R subcallosal	0.015	2.74	0.008	0.019 (0.007, 0.03)	0.129 (0.075, 0.182)	0.305 (0.068, 0.509)
L anterior cingulate	0.025	4.67	< 0.001	0.035 (0.024, 0.046)	0.178 (0.124, 0.23)	0.408 (0.184, 0.591)
R anterior cingulate	0.024	4.44	< 0.001	0.033 (0.022, 0.044)	0.169 (0.116, 0.222)	0.428 (0.207, 0.607)
L paracingulate	0.004	0.8	0.426	0.001 (− 0.01, 0.013)	0.044 (− 0.01, 0.098)	0.106 (− 0.14, 0.339)
R paracingulate	0.005	0.83	0.426	0.003 (− 0.008, 0.014)	0.052 (− 0.003, 0.106)	0.122 (− 0.124, 0.353)
Intracranial volume	0.004	0.87	0.426	− 0.009 (− 0.02, 0.002)	− 0.016 (− 0.07, 0.038)	− 0.098 (− 0.332, 0.147)

Splitting the study timeline into 6 equal periods (11 months each) we showed that the correlations are strongest during and following phase transition events, i.e. when the change and variability of stock market dynamics is most pronounced (Supplementary Fig. S8).

**Figure 3:**
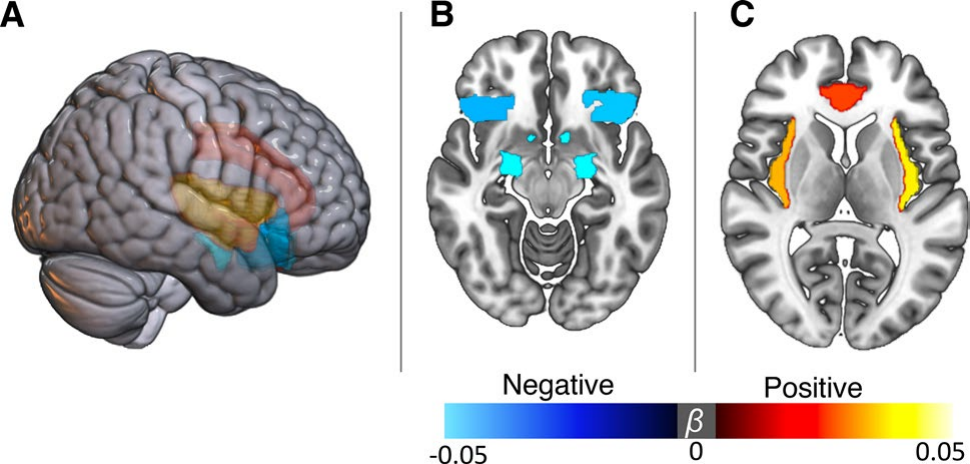
Regional profile of brain-market associations. (**A**) Three-dimensional view of the significant associations (p_FDR_ < 0.05). FTSE100 exhibited negative associations with amygdala, nucleus accumbens and orbitofrontal cortex (**B**), whereas insular and cingulate regions were positively associated with the index scores (**C**). The analyses leveraged random linear mixed effects framework with subject as a random effect, as a subset (n = 1427) of the study subjects was scanned twice.

Similar findings were observed for nucleus accumbens and lateral orbitofrontal cortex (lOFC) that also exhibited negative associations with the market (Fig. 3A,B). While nucleus accumbens is mostly known for being involved in reward anticipation, it is equally important for processing losses^15,16^. lOFC has been suggested to be involved in processing expectations within the emotional domain^36–38^, processing losses and rewards^39,40^. Further supporting this, a significant interaction (*β* = − 0.01, t_776_ = − 2.87, p = 0.004, p_fdr_ = 0.05) between FTSE100 and income index was found on the right lOFC volume (Supplementery Table S5). Post-hoc analyses revealed the highest effects in individuals with the lowest and highest income, suggesting that right lOFC of those subjects is particularly sensitive to the capital market swings. Insula and anterior cingulate cortex showed the opposite effect, i.e. the volume correlated positively with the market (Fig. 3A,C). All regions mentioned above are involved in affective processing^15–17^. The magnitude of the identified effects varied depending on time scale with median Pearson correlation *|r|*= 0.033 (0.001–0.064) for the raw data, *|r|*= 0.169 (0.017–0.282) for the day-averaged measures, and *|r|*= 0.492 (0.09–0.73) when brain and market data were averaged over months (Table 2). Importantly, all of the reported associations changed very little after detrending the FTSE100 time-series. Deconvolving FTSE100 time-series into low- and high-frequency domains using fast Fourier transform, revealed that the effect is mostly driven by low-frequency oscillations, although, a similar pattern of associations was observed for the high frequency band (Supplementary Fig. S3, Supplementary Table S6).

We amended the preregistered protocol by adding additional possible confounding variables to confirm that the main results are robust and withstand correction for age, sex, presence of psychiatric diagnoses, seasonal effects (months) and intracranial volume (Supplementary Table S3), as well as mixed generalized additive modelling^31^ conducted under various assumptions for autocorrelation structure (Supplementary Table S12). Moreover, robustness of our findings was also confirmed in the specification curve analysis^29^ that showed stability of the effects with respect to different model specification strategies (Supplementary Figs. S10, S11).

**Figure 4:**
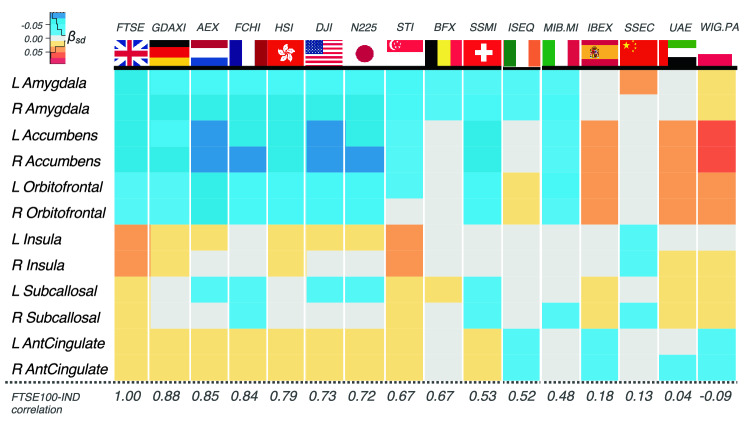
Pattern of brain-market associations for different capital market indexes. Strongest associations were found for the UK market index (FTSE100). Japanese and Singapore and Hong Kong indexes also exhibited a similar pattern of associations possibly reflecting socioeconomic and geographic similarity with the UK, whereas Dow Jones Industrial Average (DJA) likely reflects major contribution of the United States to the world economy. Chinese index (preregistered as a reference) had one of the weakest associations with the studied volumetric measures. FTSE100-IND correlations: Pearson correlation of FTSE100 with other investigated indexes. The analyses leveraged random linear mixed effects framework with subject as a random effect, as a subset (n = 1427) of the study subjects was scanned twice.

When considering the indexes of the UK’s fifteen top trading partners^41^, a similar pattern of associations to the one for FTSE100 was observed for the equivalent local European indexes (e.g. German GDAXI, Dutch AEX, French FCHI) but was of smaller magnitude (Fig. 4). The associations further declined or had different directions for markets that were more distant in a socioeconomic dimension (as also reflected in a weaker correlation with FTSE100), including the reference Shanghai Composite Index (SSEC). Importantly, the results also withstood correction for these indexes (Supplementary Table S4), which implies that the local economic performance captured by the FTSE100 exhibits a specific association with the characteristics of the scanned UK population.

Regarding causality, the most widely accepted hypothesis states that population mood and well-being are impacted by market via effects on the socioeconomic environment^4,6,28^. The most simplistic (and probably naïve) interpretation is that these effects exhibit influence on changes in housing prices^42^ and unemployment rates^43^, which, in turn, can be perceived as threat signals that impact brains and emotional states of the population^10^. Another hypothesis stemming from socionomics is currently growing in popularity. It puts forward the idea of “social mood” as a herding-driven emergent state that originates from population dynamics and subsequently drives global processes, including economic crises, wars, art and fashion^1,2^. According to this hypothesis, social mood is an inherently hidden state of the society. It is related (but not identical) to the mood of individuals that such a group consists of. This hypothesis is conceptually supported by the data acquired in small-scale experimental studies demonstrating involvement of reward and fear circuits in future financial decisions^44–46^. Of importance for the present discussion, this hypothesis considers stock market dynamics as a valuable “metric stick” of the social mood and global societal dynamics^1^.

**Figure 5:**
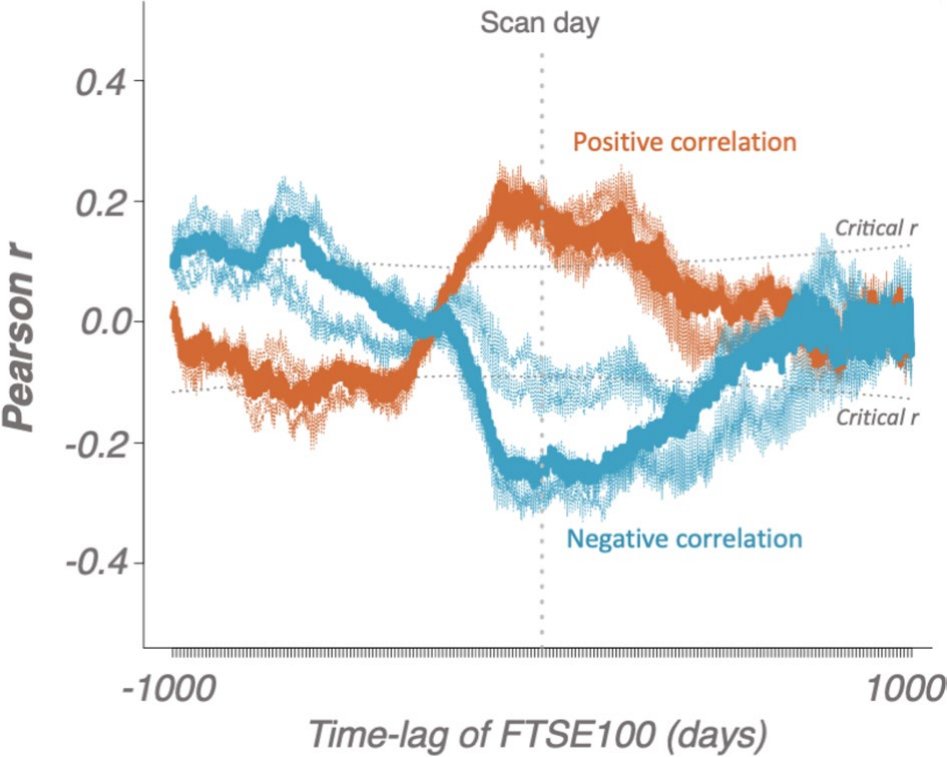
Pearson correlations for the brain and FTSE100-lagged data averaged over days. Transparent lines represent individual regions whereas thick lines represent medians of the correlations. Dotted boundaries represent critical r-values for α = 0.001. The plot represents magnitudes of associations between brain data at the date of scanning and the FTSE100 index shifted forward (right) and backward (left) in time. Note a reversed peak for earlier dates reflective of autocorrelations.

To begin to further investigate these relationships, we evaluated associations with time-lagged Pearson correlation. We identified that brain volumes correlate higher with *earlier* market prices. The correlation remains significant for approximately one year and then gradually decays (Fig. 5). While an autocorrelation, as expected, is present in the stock market time-series^47^ (Supplementary Table S7), the fact that earlier economic data peaks with the brain volume implies that the market events may be antecedent to the brain volume fluctuations, offering initial evidence that the market “impacts” the brain, mood, and well-being. The same analyses were carried out on the monthly scale yielding similar results (Supplementary Fig. S4) and also for the mood data with the FTSE100, although no clear antecedent relationship could be drawn for the latter (Supplementary Fig. S5).

**Figure 6:**
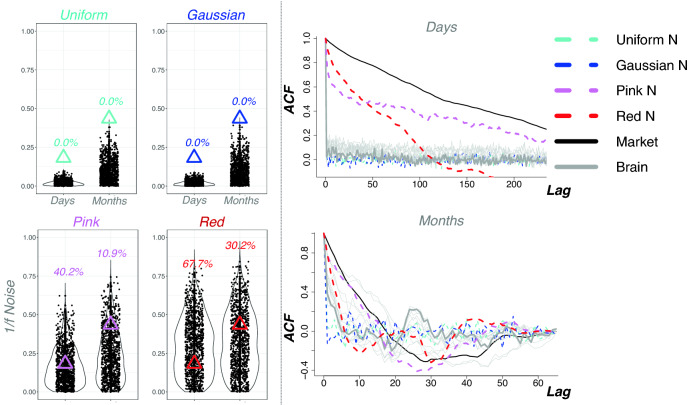
Noise simulation experiments and autocorrelation function density plots. Left: Uniform and gaussian noise simulations failed to produce the effect sizes of equivalent (root-squared) magnitude to the one found in the present study (top). However, 1/f noise was capable of inducing such associations (bottom). Note that we intentionally used root-squared estimates to illustrate these effects. Without this step, all of the estimates from multiple noise simulations converge to zero (Supplementary Fig. S12), unlike the reported results showing consistent directionality in different time-bins and three independent samples. Right: Autocorrelation function (ACF) density plots demonstrating scale-free properties of the stock market data most similar to the ones of 1/f noise (pink and red).

We then leveraged Toda-Yamamoto implementation of Granger Causality for non-stationary data^33^ to numerically test two competing models that characterize “brain-market” associations. Despite the fact this procedure specifically designed for serially correlated data provided somewhat stronger support in favour of a causal link “Market impacts Population Brain/Mood, it is worth noting that the opposite hypothesis could not be completely rejected for amygdalae and subcallosal cortex (Supplementary Tables S8 and S9). An extra caution is also advised when interpreting these analyses due to scale-free properties of the investigated time-series (Fig. 6). To illustrate this point, we first show the absence of any significant effects after shuffling the dates (Supplementary Table S10, column 1), but appearance of residual associations for the time-shifted data (Supplementary Table S10, column 2, also seen on Fig. 5). Importantly, simulating stock market data with 1/f noise is capable of producing effect-sizes of similar magnitude (Supplementary Table S10, column 3), pooled effect of which, however, converges to zero due to inconsistency of directions in the estimated associations (Supplementary Fig. S12), and, unlike the main results, also disappear after adjusting for other stock market indexes (Supplementary Table S10, column 4), confirming that the main effect is not driven by a randomly-seeded 1/f noise. Moreover, we demonstrated that the magnitude of the brain-market links (measured as median squared root correlations) is related to economic and sociocultural ties of the UK to other countries^30,41^ (Supplementary Figs. S6 and S7) and that no other global candidate metrics with 1/f properties (UK seismic activity and mortality rates) exhibit an equivalent level of specificity with respect to the investigated variables (Supplementary Fig. S9).

Due to self-similarity properties identified in the data (Fig. 6, right panel), we decided to conduct a follow-up series of noise simulation experiments. Simulating brain data with uniform and gaussian noise failed to induce the afore-mentioned correlations with FTSE100, but, as expected, they were more likely to be discovered for the brain data simulated with 1/f noise (Fig. 6, left panel).

Therefore, it appears so that scale-free properties are observed at different levels of population dynamics, which is reflected in fluctuations of stock markets, mood and brains. To confirm that the effects still hold after accounting for scale-free noise, we repeated the simulations of brain data with 1/f noise and matched it with the stock markets of the UK’s 15 top trading partners. We then subtracted the yielded Pearson correlations from the real ones (prior to calculating the medians) and, as expected, the effect sizes only became larger (Supplementary Fig. S7B). Moreover, a negative association was also identified for a number of sociocultural distances of the UK from 17 countries using data from Liu et al.^30^ (Supplementary Fig. S7). All of the above supported the hypothesis of the stock market as a useful metric stick for global societal dynamics^48^.

## Discussion

Our study was motivated by previous findings demonstrating significant relationships between economic indicators and population well-being, particularly evident in turbulent times^1–9^. Our study is the first to show that both mood and brain are highly sensitive to the bellwether of societal change, namely the stock market. The amended study protocol employed a wide variety of strategies to confirm the discovered associations. Our imaging and non-imaging results withstood adjustment for linear and non-linear effects of time and were replicated employing mutual information criterion. We also used two independent datasets from the United States to replicate the findings. Furthermore, we conducted a number of follow-up analyses demonstrating specificity and convergent validity of the results in relation to socioeconomic and cultural distances. Finally, after identifying scale-free properties in the data we also conducted a series of simulation experiments demonstrating that effects of similar magnitude can be induced by simulating the data with 1/f noise, polled effects of which, however, unlike the main results, converged to zero. As expected, adjusting our results for scale-free noise only boosted the investigated effect-sizes.

There is a number of important considerations that must be taken into account when interpreting our results. First, violation of random sampling assumptions can potentially occur in large-scale datasets collected over long time-periods, including the UK Biobank data^49^. In the present study, sampling bias may be present due to absence of explicit stratification for participants’ socioeconomic status, which, in turn, may influence probability to volunteer in different socioeconomic environments. It is worth noting, however, that our results survived all of the undertaken adjustments for potential analytical biases and potential confounds, including research site, age, sex, linear and non-linear effects of time, patient status, other stock market indexes, intracranial volume, as well as all possible combinations of the selected confounds in the specification curve analyses^29^. However, if these assumptions are, indeed, consistently violated across different time-bins and dataset scales, resulting in the same effects in different samples, including longitudinal single-subject studies, this is already an important finding implying that the investigated effects are big enough to impact complex behavioural patterns, including enrolment likelihood of individuals with certain psychological and biological traits, or at the very least represent an important confound that must be taken into account when conducting meta-analyses or designing any studies (cross-sectional or longitudinal) that use data collected over long periods^34^. Specifically, it would be interesting to investigate to what extent socioeconomic environment contributes to reproducibility crises in medicine and psychology^50,51^. The same caution applies to potential presence of scanner drifts. And whilst it is theoretically possible for them to uniquely exhibit similar dynamics to the one of stock markets, we address this limitation by showing similar associations with the non-MRI (behavioural) measures, suggesting that these effects generalize to different types of data.

Another important point of discussion is the topic of randomness and the origin of scale-free noise in complex systems. Modern studies suggest that the stock market behaviour should not be modelled as a ‘random walk’ (i.e. having Gaussian distribution), but rather as a non-Gaussian process with random ‘jumps’ resulting in fat-tailed distributions^32^. In such cases, leveraging linear methods to estimate associations between two variables may not represent the best solution. Recognizing importance of this point we replicated our results using mutual information criterion, which, as some may argue, may be a more potent strategy for addressing the afore-mentioned non-linear effects.

It is also worth noting that the discovered effects appeared to be substantially larger than we initially expected. This is reflected in the reported results of the whole-brain analyses that demonstrated effects in regions that are not directly involved in emotional processing. However, our whole-brain findings still largely overlap with the regional profile reported by Salomon et al.^14^ demonstrating (besides previously mentioned amygdalar changes) volumetric increases in putamen and the anterior temporal cortex, which the authors linked to the intense experience associated with the COVID19 pandemic.

Finally, we would like to highlight again that the investigated market variable (FTSE100 index) does not represent the stock market per se, but rather reflects a current socioeconomic state of the society. This metric itself can therefore be the subject of economic biases, affecting global valuation of stocks. In light of this, future studies may want to explore effects of quantitative easing and tightening on the studied indexes and consider adjusting the investigated effects for total money supply upon occurrence of such events.

## Conclusion

Thus, the main preregistered hypothesis that large-scale societal dynamics are related to neuromorphological characteristics of the studied population has been confirmed in several ways. Our results provide novel and strong evidence that even if we are unaware of the state of the stock market, it is still an important indicator of societal change, which, in turn, is related to our well-being or, at the very least, to the structure of well-being-related data acquired over long time periods. Taken together, our results provided evidence for self-organized criticality present in stock market behaviour supporting the socionomic hypothesis of “social mood” as a driving factor in global societal processes. Here we show that these dynamics may originate in scale-free behaviour occurring in many complex systems in nature^47^. Despite being small on an individual level, these effects may have a large influence on a population level, as the previous studies have suggested^4–8,10^. This is underscored by our objective measure of diastolic blood pressure that on average differed five units between the samples measured during the lowest market outcomes compared with the ones measured during the highest market outcomes. This effect may have clinical relevance on a population level. Moreover, our results suggest that some sub-populations are particularly vulnerable to economic turbulences, such as individuals with low and very high income. Understanding these complex but nevertheless important processes is of crucial relevance for sustainable and well-being-oriented economic development^52,53^.

## Supplementary Information

Supplementary Information.

## Data Availability

The access to the UK Biobank data was granted to the authors after submitting project description with stated hypotheses and analysis plan. The study was preregistered at the Open Science Foundation Framework database (https://osf.io/h52gk) prior to data transfer. UK Biobank remains the owner of the database and accepts data request from third parties after approving corresponding project proposals and payments of the data access fees (more details: https://www.ukbiobank.ac.uk/principles-of-access). Similarly, Parkinson’s Progression Markers Initiative MRI data used to replicate the main findings is not publicly open, but can be accessed after completing a corresponding registration form (https://www.ppmi-info.org). Main data analysis steps are illustrated with MyConnectome data in a reproducible R-script attached to the submission (“GETAB_Poldrack.R”).
