# Author Correction: Large-scale societal dynamics are reflected in human mood and brain

**DOI:** 10.1038/s41598-022-10445-z

**Published:** 2022-04-13

**Authors:** Alexander V. Lebedev, Christoph Abé, Kasim Acar, Gustavo Deco, Morten L. Kringelbach, Martin Ingvar, Predrag Petrovic

**Affiliations:** 1 Department of Clinical Neuroscience, Karolinska Institutet, Stockholm, Sweden; 2 Center for Cognitive and Computational Neurosceince (CCNP), Karolinska Institutet, Stockholm, Sweden; 3 Center for Brain and Cognition, Computational Neuroscience Group, Department of Information and Communication Technologies, Universitat Pompeu Fabra, Barcelona, Spain; 4 Institució Catalana de la Recerca i Estudis Avançats (ICREA), Barcelona, Spain; 5 Centre for Eudaimonia and Human Flourishing, Linacre College, University of Oxford, Oxford, UK; 6 Department of Psychiatry, University of Oxford, Oxford, UK; 7 Center for Music in the Brain, Aarhus University, Aarhus, Denmark

Correction to: *Scientific Reports* https://doi.org/10.1038/s41598-022-08569-3, published online 17 March 2022

The original version of this Article contained errors.

The authors omitted the below Reference, which is listed below as Reference 48.

48. Prechter, R. The Wave Principle of Human Social Behavior and the New Science of Socionomics, New Classics Library. ISBN-10:0932750494, (1999).

As a result, in the last sentence of the last paragraph in the Results section,

“All of the above supported Casti’s hypothesis of stock markets as a useful metric stick for global societal dynamics^1^.”

now reads:

“All of the above supported the hypothesis of the stock market as a useful metric stick for global societal dynamics^48^.”

As a result of this change, the References have been renumbered.

The original Article has been corrected.

