# Supplementary material for: Large-scale societal dynamics are reflected in human mood and brain
Source: Sci Rep. 2022 Mar 17;12:4646. doi: 10.1038/s41598-022-08569-3 (PMC8931098; doi:10.1038/s41598-022-08569-3)
Supplement: Supplementary file 1 — Supplementary Information. [file 41598_2022_8569_MOESM1_ESM.pdf]

Supplementary Materials

Materials and Methods

Preregistration

The study was conducted in accordance with the Declaration of Helsinki on Ethical Principles for Medical Research Involving Human Subjects [1] and after approval of the submitted research proposal by the UK Biobank (Application ID #62895) was preregistered at the Open Science Foundation Framework database (https://osf.io/h52gk) prior to data transfer. The primary submitted hypothesis was that "global market fluctuations exhibit significant associations with structure and function of brain regions of the fear circuit". The present set of analyses was fully focused on the structural data.

Subjects

The current project targeted a population of British citizens from the UK Biobank. The main sample consisted of 41,182 data-points from a total of 39,755 UK citizens who completed MRI sessions at least once and was assessed over a period of approximately 4.5 years (between 2014-05-02 and 2019-10-31). The larger sample consisted of 547,005 data-points (479,791 individuals) and was used in the analyses of mood-market relationships (See **Table S1** for descriptive statistics of the two samples).

MRI data

Brain scans were collected on a 3 Tesla scanner Siemens Magnetom Skyra Syngo MR D13. Structural T1 3D scans were collected adhering to a standardized protocol as described in the UK Biobank materials (see https://www.fmrib.ox.ac.uk/ukbiobank/protocol/index.html): TA: 4:54, voxel size:1.0×1.0×1.0 mm.

Structural scans were preprocessed employing automated steps as implemented in FSL, yielding region-specific measures of cortical and subcortical volumes parcellated according to the Harvard-Oxford atlas (https://fsl.fmrib.ox.ac.uk/fsl/fslwiki/Atlases).

The main analysis was focused on 14 regions-of-interest that were selected in advance as the ones playing major roles in affective processing: amygdala, nucleus accumbens, insula, anterior, subcallosal and dorsal cingulate and lateral orbitofrontal cortical areas.

Market Data

Historical daily time-series data of FTSE100 stock market index was extracted from yahoo finance website (https://finance.yahoo.com/) and matched with fluctuations of structural brain measures of the studied population on each of the scanning days.

The preregistered market outcome was The Financial Times Stock Exchange 100 Index (FTSE100), a widely accepted metric of UK economic performance characterizing stock price of the top 100 UK companies with biggest revenue.

For the analyses we used the daily adjusted close value that represents the closing price after adjustments for all applicable splits and dividend distributions adhering to Center for Research in Security Prices (CRSP) calculation standards. No additional transformations were applied on the extracted time-series in the results reported in the main text. However, the stability of the investigated associations was later confirmed on the de-trended time-series. As a part of the exploratory analyses, we also looked into low- and high-frequency bands of the market time-series deconvolved with fast Fourier transform.

Analysis

Initially, we planned to employ classical regression methods to estimate linear relationships between brain and stock market data, followed by a correction for potential confounds. However, after discovering that we will receive access to subjects that were scanned twice (n=1427) we decided to take advantage of this by employing methods of linear mixed-effects modelling introducing "subject" as a random effect variable. All of the main analytical steps, however, were further repeated with classical regression methods confirming the presented results.

Causality tests were applied on the primary outcomes to investigate two alternative causal paths of the studied processes: H1: "brain impacts market" and H2: "market impacts brain".

The workflow adhered to the Toda-Yamamoto implementation of Granger Causality for non-stationary data [2] and consisted of: 1) testing for integration and determining max order of integration, 2) setting up a VAR-model in levels for the non-differenced data, 3) determining the lag length, 4) Portmanteau test for residual serial correlation, 5) adding the maximum order of integration to the number of lags, generating the augmented VAR-model, 6) application of the Wald χ^2 test of the two alternative augmented models: "market impacts brain" and "brain impacts market".

All statistical analyses were performed using R programming language. Linear mixed-effects models and Toda-Yamamoto Granger causality analyses were implemented using ‘nlme’ and ‘vars’ packages, respectively.

Figures

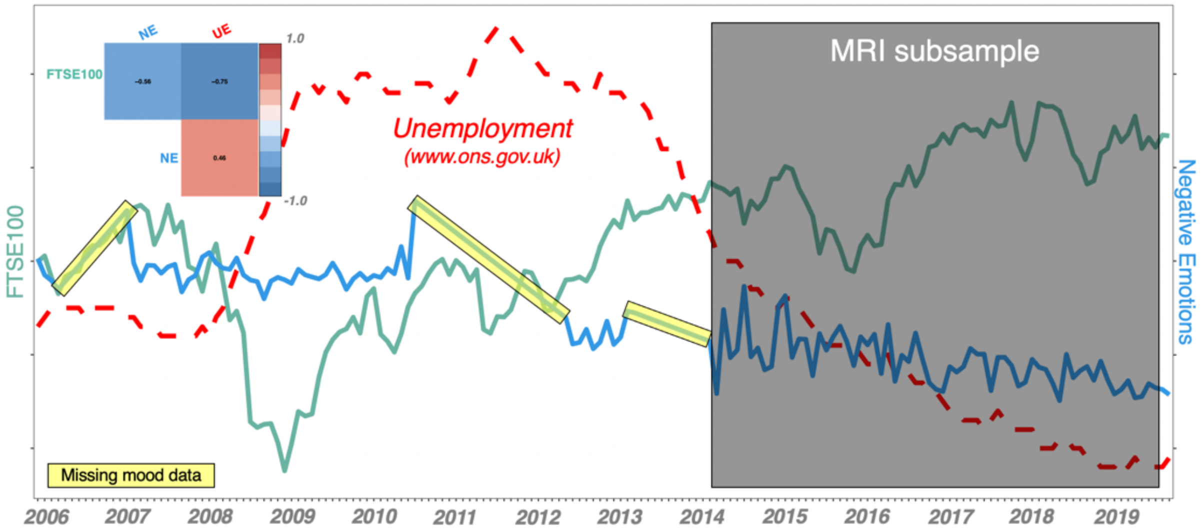

Fig. S1*. *Illustration of the investigated period. *Mood data was available for a larger period of approximately 14 years and was missing for a number of time points (interpolated on the figure, but not in the main analyses). The plot illustrates dynamics of the UK stock market (FTSE100), unemployment levels (UE, source:* http://www.ons.gov.uk *and self-reported negative emotions (NE) for this period. MRI data was available for a period of approximately 5.5 years.*

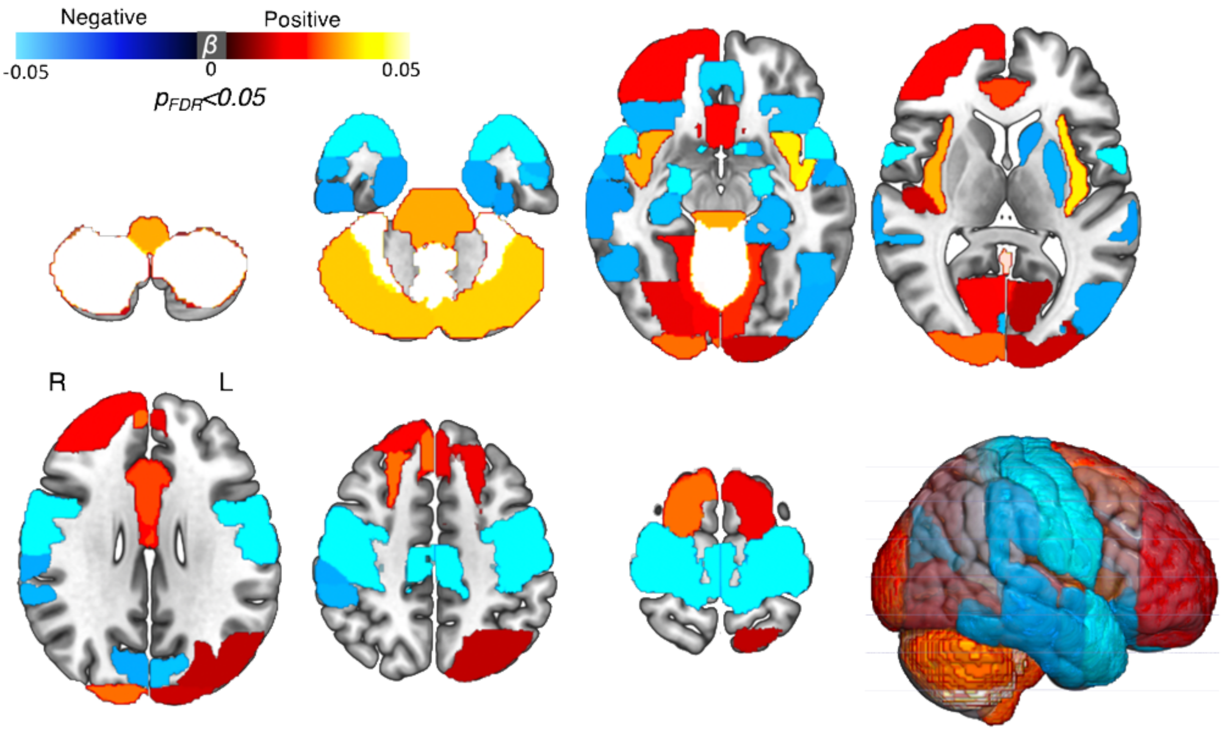

Fig. S2. Associations between FTSE100 and grey matter volumes*: whole-brain analysis. The associations were estimated employing mass-univariate strategy following correction for multiple testing with false discovery rate. Whilst the effects were not specific to the selected regions that are proven components of the fear/reward network, it can be noted that the largest effects are, indeed, seen in the areas playing key roles in affective and motor processing.*

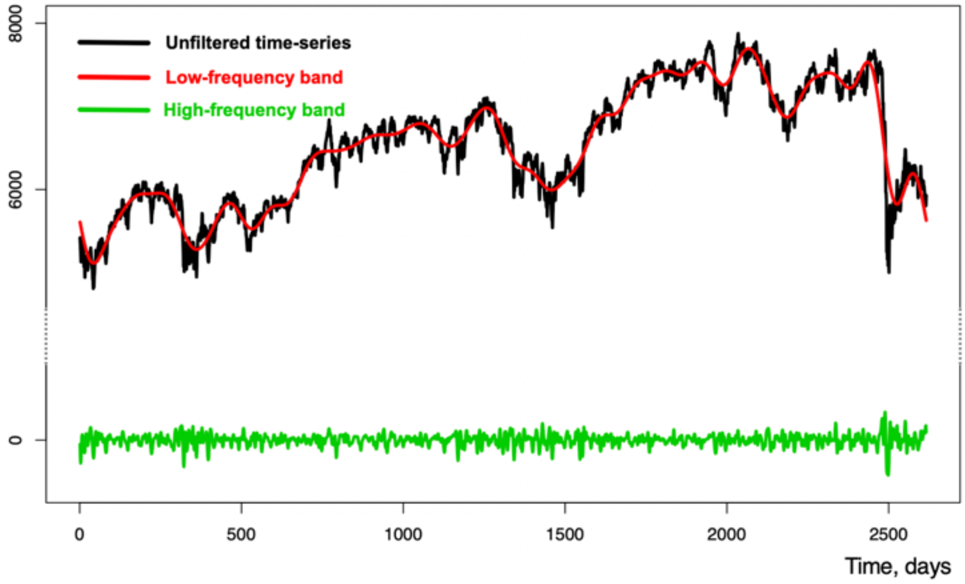

Fig. S3. Frequency band analysis. *FTSE100 time-series deconvolved with Fast Fourier Transform (FFT) into low- and high-frequency bands, which were further analyzed in relation to the brain and mood data employing methods of linear modeling.*

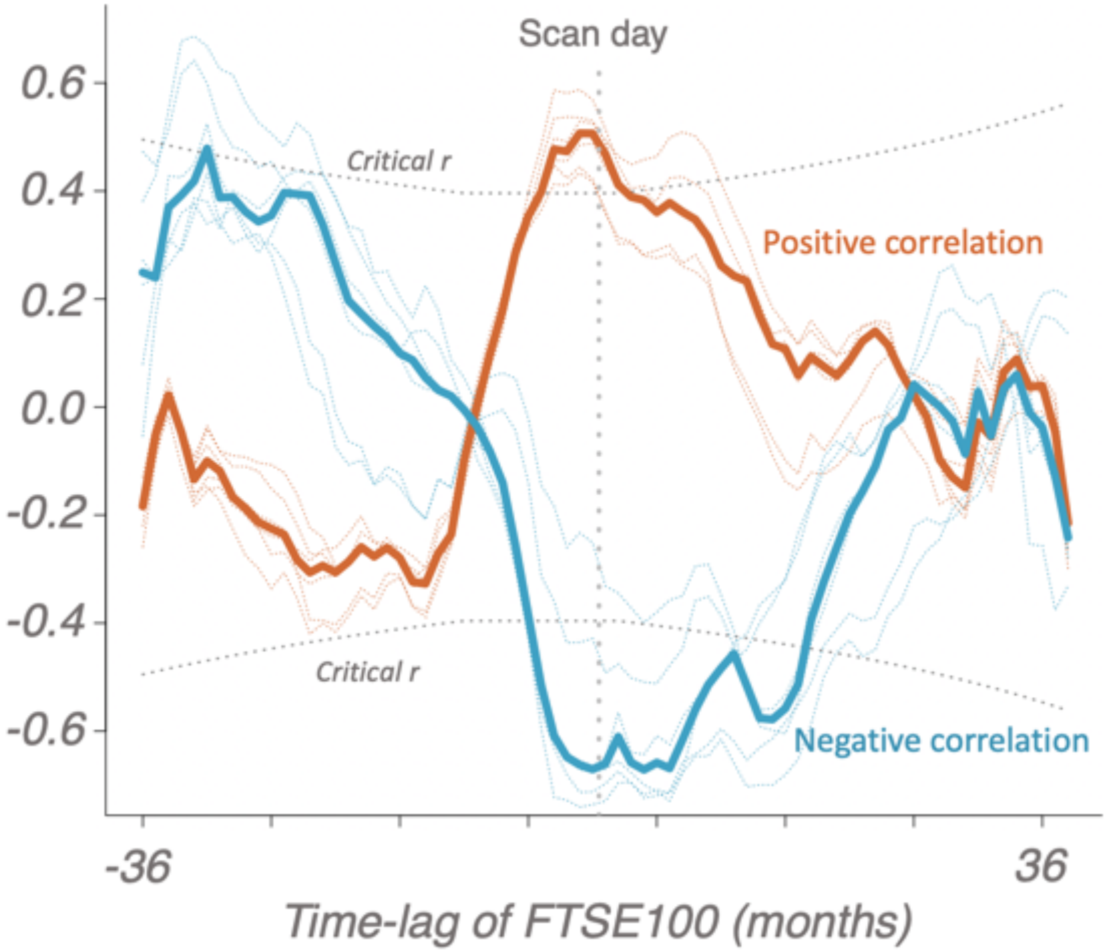

Figure S4. Pearson correlations for the brain and FTSE100-lagged data averaged over months. *Transparent lines represent individual regions whereas thick lines represent medians of the correlations. Dotted boundaries represent critical r-values for α=0.001.*

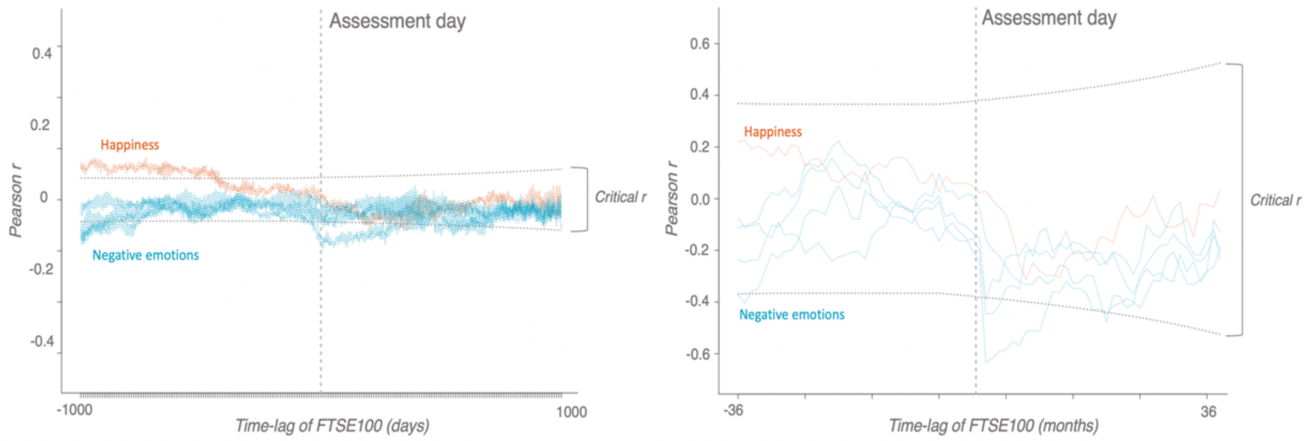

Figure S5. Correlations over days and months. *Pearson correlations for the mood and FTSE100-lagged data averaged over days (left) and months (right). Dotted boundaries represent critical r-values for α=0.001.*

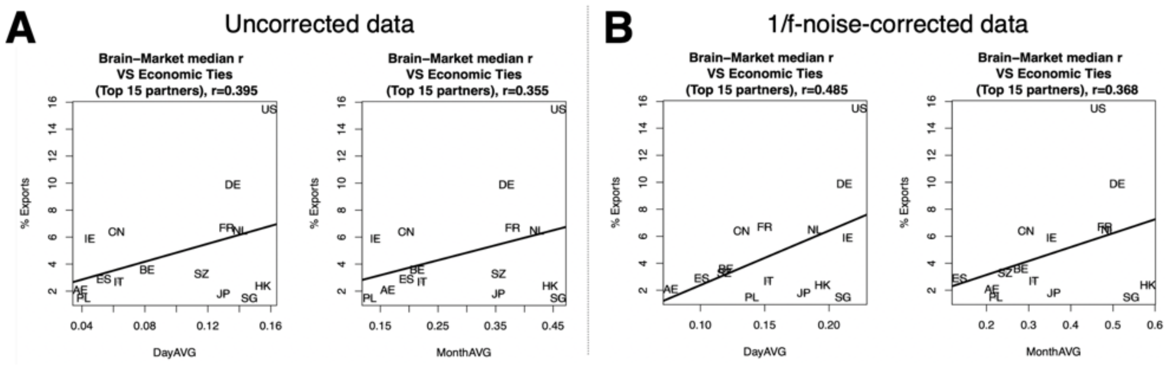

Figure S6*. *Association between Brain-Market link and economic ties with 15 UK's top trading partners. *The brain-market link measured as median Pearson correlation (r) for all of the 12 regions that exhibited significant associations in the main analysis was matched with markets of 15 UK's top trading partners. The strength of economic ties was measured as a relative percent of all exports accessed from www.worldstopexports.com. Here we report the associations for the raw brain-market links.* ***A****), as well as the results following correction for 1/f noise.* ***B****), which entailed 1) simulation of the brain data with pink noise and 2) subtraction of the yielded correlations from the real data prior to calculating the medians. As expected, this procedure slightly increased the effect-sizes by increasing signal-to-noise ratio. Country labels: BE – Belgium, CN – China, FR – France, DE – Germany, HK – Hong Kong, IE – Ireland, IT – Italy, JP – Japan, NL – Netherlands, PL – Poland, SG – Singapore, ES – Spain, AE – United Arab Emirates, US – United States.*

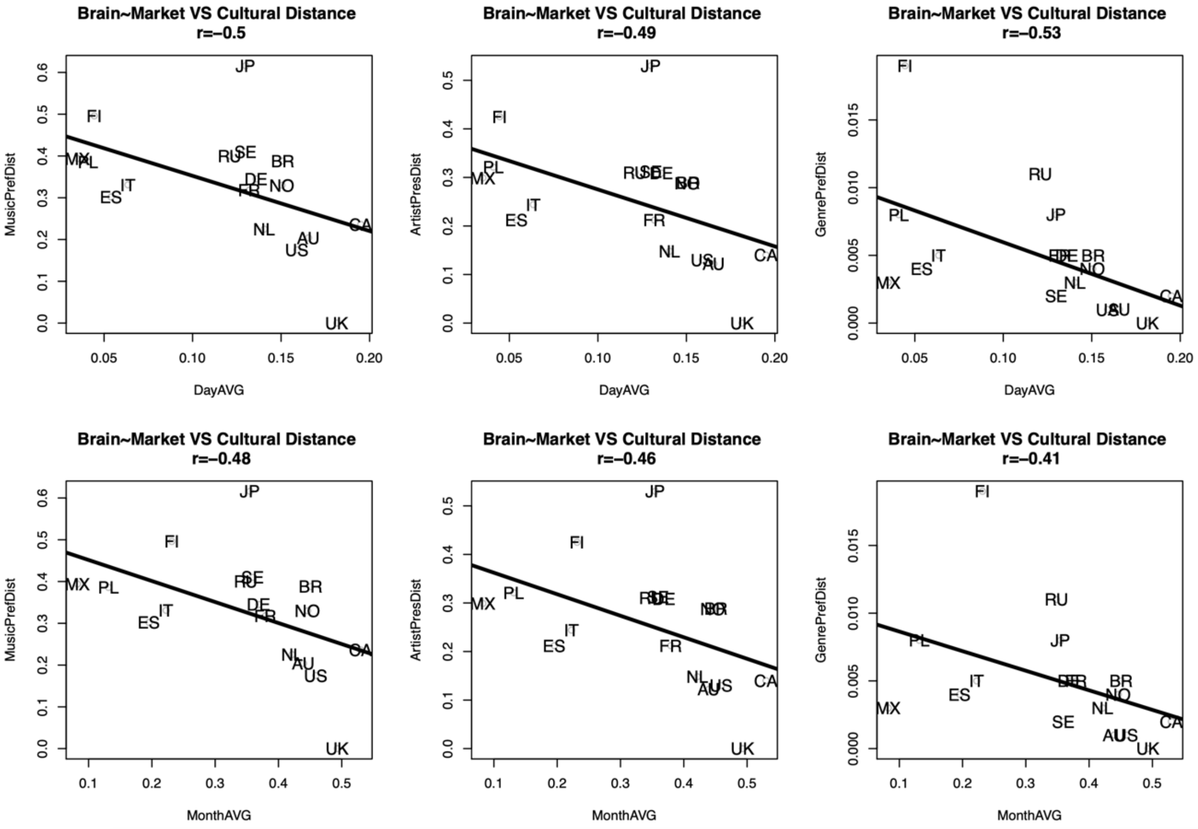

***Figure S7**. **Association between Brain-Market link and sociocultural distances of the UK from 17 countries using data from Liu et al, 2018** [3]. The brain-market link was measured as median Pearson correlation (r) for all of the 12 regions that exhibited significant associations in the main analysis. The sociocultural distance was calculated by Liu et al based on music (MusicPrefDist), artist (ArtistPrefDist) and genre preferences (MusicPrefDist). A negative relationship was found for the Brain-Market link and the afore-mentioned distances, i.e. the stronger the link the shorter the distance. The original study was focused on the following 20 countries: the United States (US), Russia (RU), Germany (DE), the United Kingdom (UK), Poland (PL), Brazil (BR), Finland (FI), Netherlands (NL), Spain (ES), Sweden (SE), Ukraine (market data not available), Canada (CA), France (FR), Australia (AU), Italy (IT), Japan (JP), Norway (NO), Mexico (MX), Czech Republic (market data not available), and Belarus (market data not available). The genre labels are artist-based and correspond to those in Allmusic, a major online music repository.*

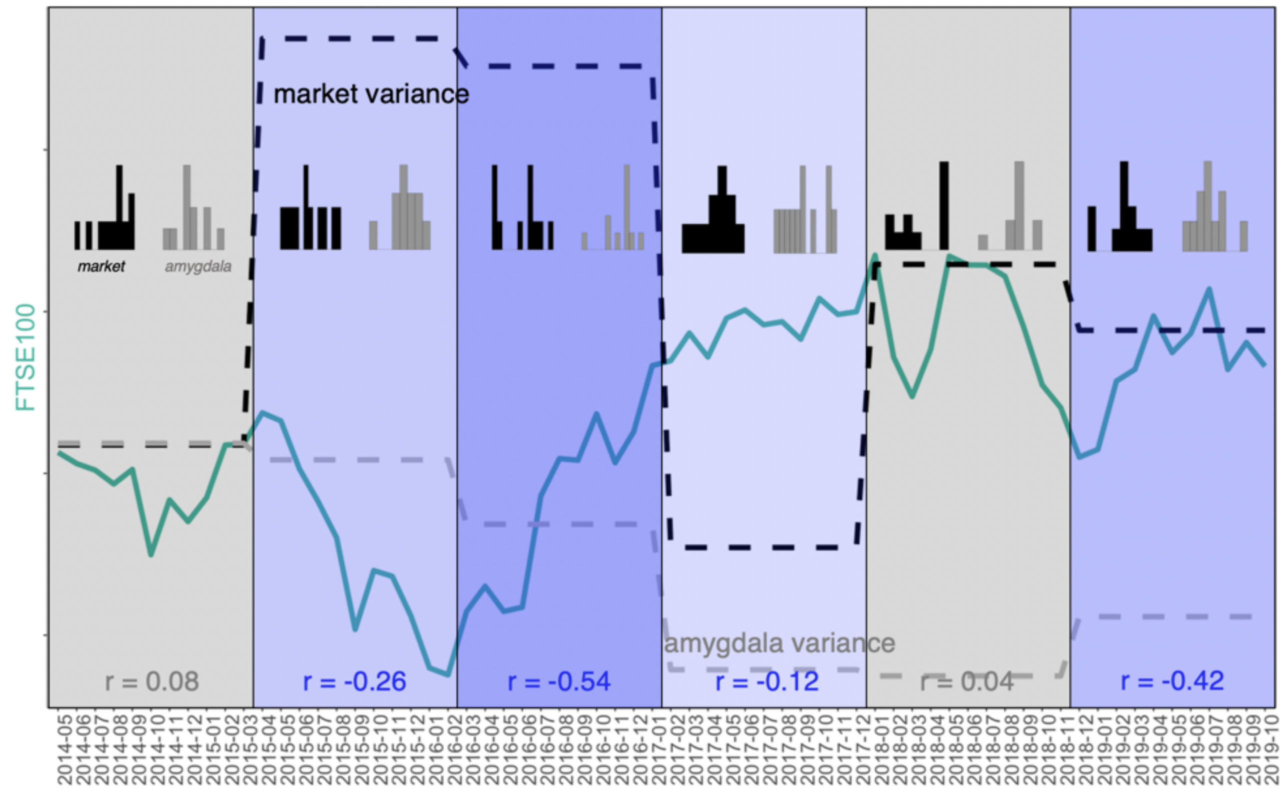

Figure S8. Correlation between amygdala volume and FTSE100 in different study periods. *The timeline was split into 6 equal bins (11 months each) and correlations were calculated for each bin separately. The figure shows that the correlations are strongest during and following phase transition events, i.e. when the change and variability of stock market dynamics is most pronounced.*

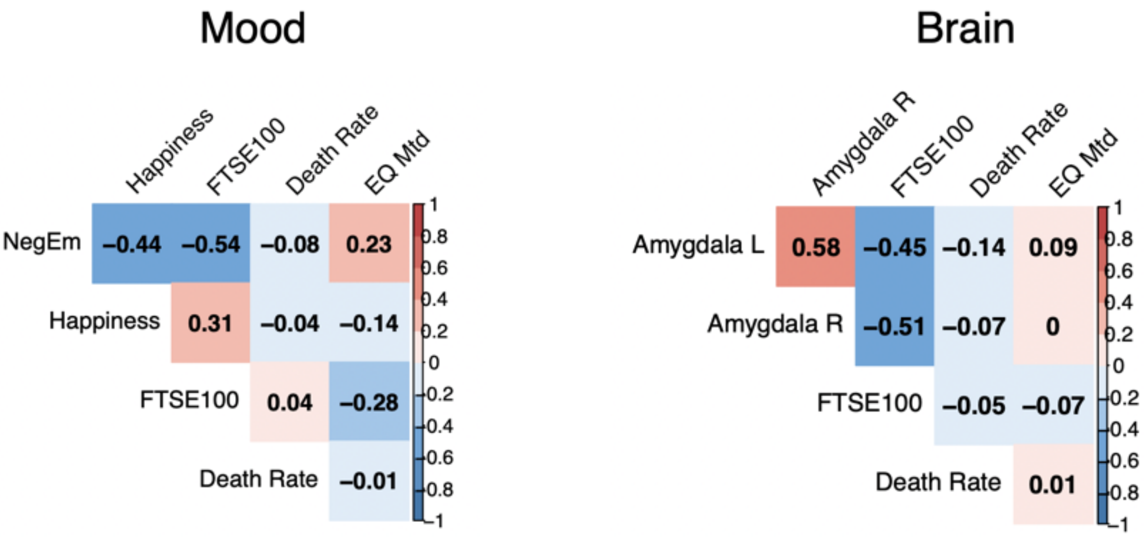

Figure S9. FTSE100 and alternative global candidate metrics with 1/f properties*. The correlation plots show that compared to other global metrics FTSE100 exhibited strongest association with the investigated variables. All time-series were converted to a weekly scale for consistency (original scale of the public UK mortality data from www.mortality.org). Public UK seismic activity data measured on Richter magnitude scale (EQ Mtd) was accessed via earthquakes.bgs.ac.uk.*

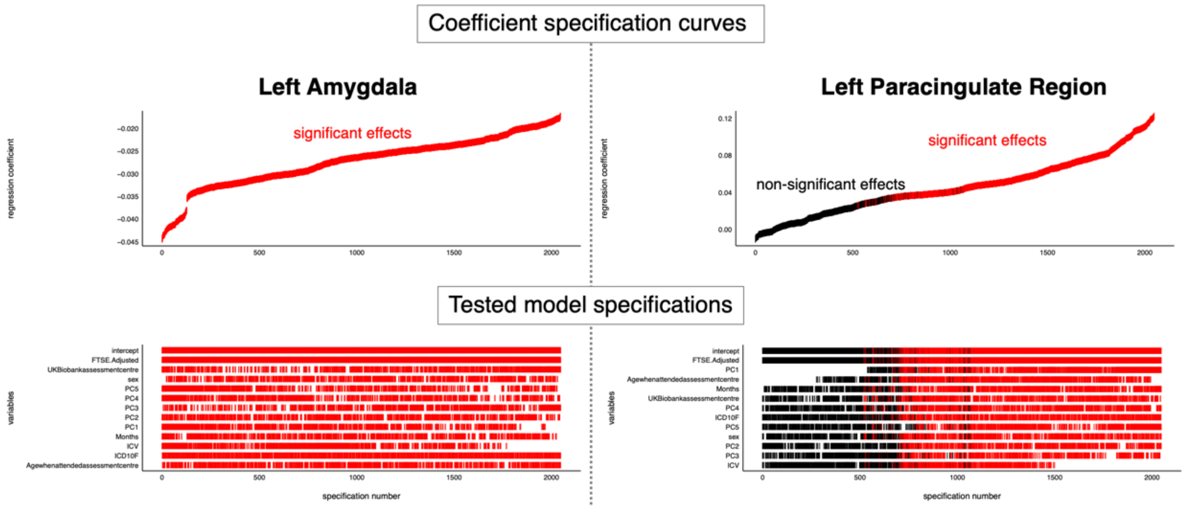

Figure S10. Specification curve analysis of the most and least significant brain regions from the main results. *The analysis adhered to the Simonsohn's protocol [4] focusing on the brain regions that had the strongest (amygdala) and weakest (paracingulate cortex) association with the FTSE100 index. The protocol entailed: 1) specification all reasonable models (to introduce all of the investigated nuisance covariates and their combinations), 2) plotting specification curves showing estimates/model fits as a function of analytic decisions, 3) testing how consistent the curve results are against a null hypothesis. The plot shows robustness of the investigated effects with respect to a wide variety of model specification strategies (i.e. most of the covariate combinations resulted in statistically significant estimates). All of the tested models fitted the data substantially better than a null model according to the AIC-criterion except for the one specifying non-adjusted effects of the FTSE100 on the paracingulate region, which, in line with the reported results, was equivalent to the null model (intercept only). All possible nested models were generated using the dredge() function from the MuMIn package.*

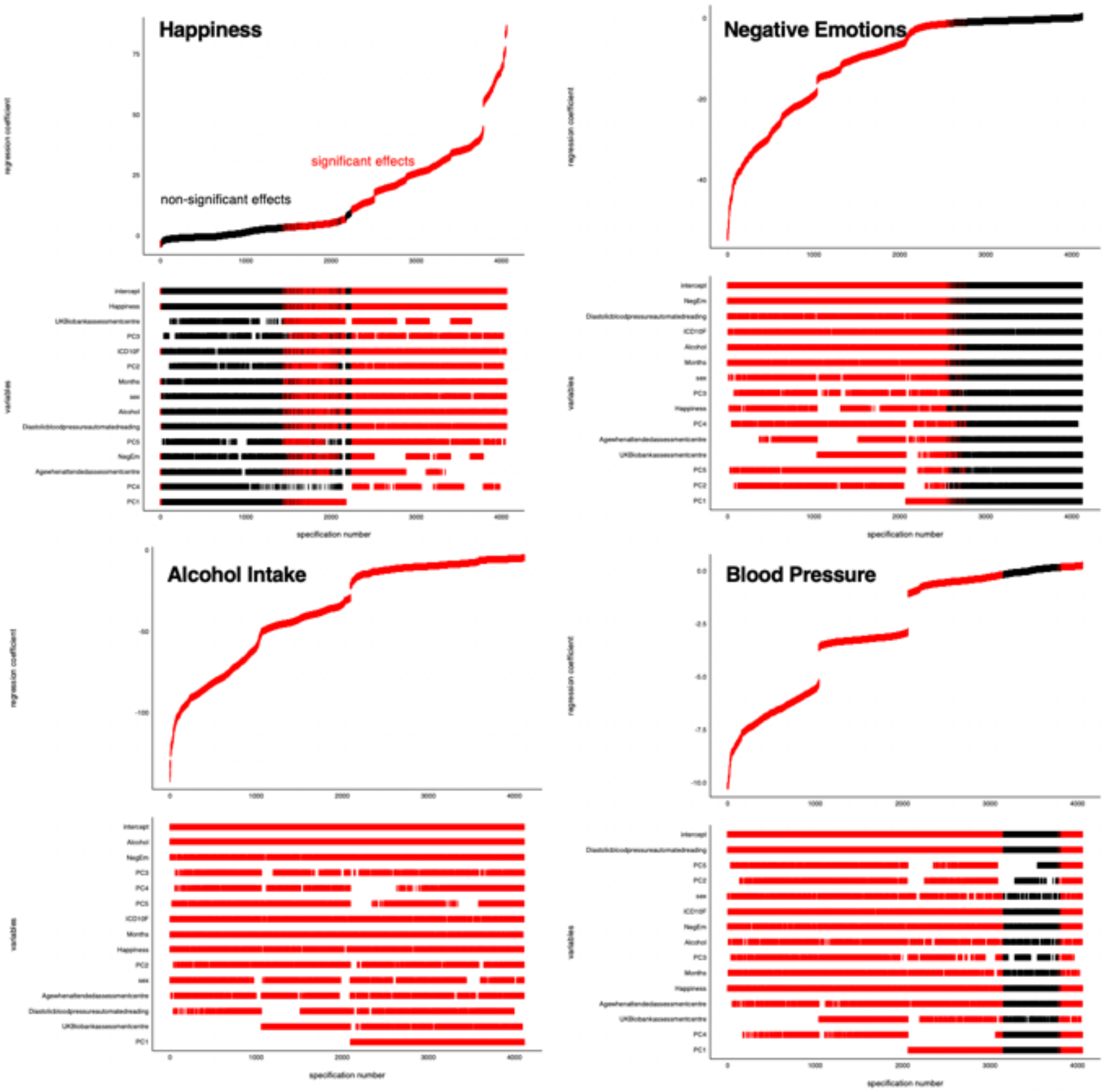

Figure S11*. *Specification curve analysis of the associations between FTSE100 and all of the investigated non-MRI variables: 14 years. *For the present analysis FTSE100 was specified as a dependent variable in order to investigate independent variance contribution of all of the investigated non-MRI variables-of-interest (happiness, negative emotions, alcohol intake and diastolic blood pressure) and a number of confounds (non-UK stock markets, age, sex, psychiatric diagnosis, assessment center, seasonal effects) for the extended (14-year) period of the study. The plot shows stability of the investigated effects. All of the tested models fitted the data substantially better than a null model according to the AIC-criterion. A random 50% sample of all possible nested models were generated using the dredge() function from the MuMIn package.*

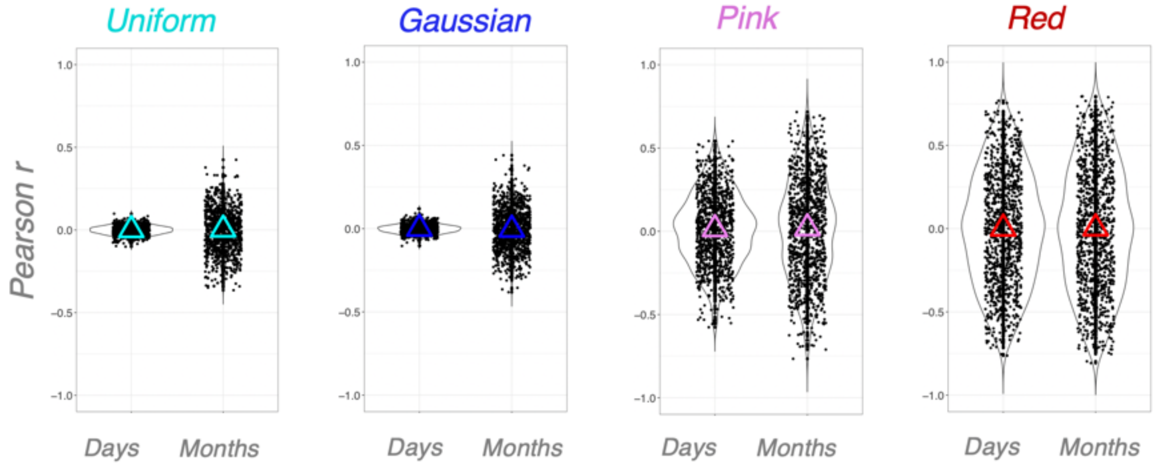

***Figure S12*. Raw noise simulation experiments.**
The plot shows convergence of the estimated effect-sizes to zero when no rootsquared-transformation is applied.

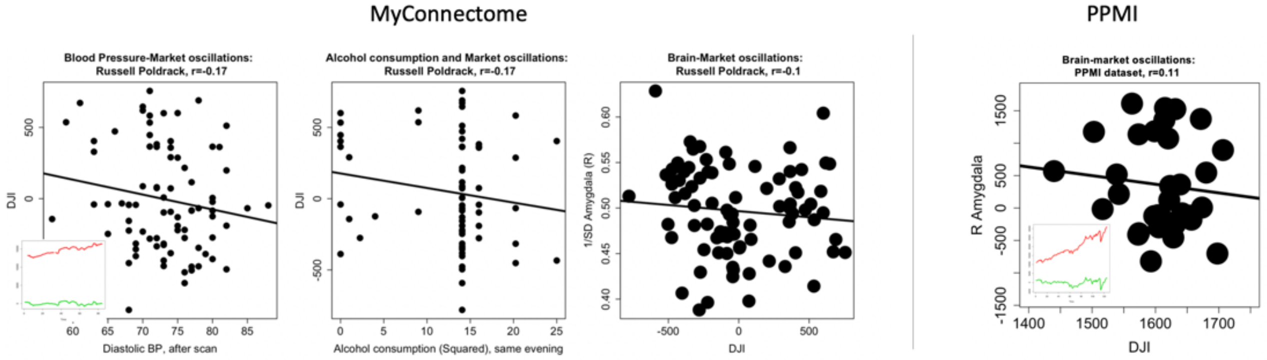

***Figure S13*. Detrended results in the MyConnectome and PPMI datasets.**
The plots demonstrate substantial reduction of effect-sizes with sustained directionality of the associations.

Tables

	**Mood**	**Brain (main)**
**Period, years (span)**	2006-03-13 - 2020-03-13	2014-05-02 - 2019-10-31
**Unique subjects / N, total**	479,791/ 547,005	39,755/41,182
**Age, years**	57.39(±8.35)	63.67(±7.54)
**Sex, Males/Females, %Males**	251635/295370 I 46% M	19434/21748 I 47.19% M
**Education, years**	16.43(±3.31)	16.97(±2.63)
**Intelligence score***	6.17(±2.14)	6.63(±2.06)

** Unweighted sum of the number of correct answers given to the 13 fluid intelligence questions.*

Table S1. Study samples: descriptive statistics

	β_{std}	*T(df)*	p_{fdr}	*Raw*	*DayAVG*	*MonthAVG*
**Negative Emotions (total)**	-<br>0.09	-<br>27.85(7905)	<0.001	-0.106(-0.113,-0.099)	-0.23(-0.275,-0.185)	-0.598(-0.717,-0.445)

Irritability	-<br>0.03	-7.58(7905)	<0.001	-	-0.068(-0.116,-0.021)	-0.202(-0.394,0.006)
Sensitivity/hurt feelings	-<br>0.09	-<br>25.51(7905)	<0.001	-	-0.233(-0.277,-0.187)	-0.467(-0.616,-0.287)
Nervous feelings	-<br>0.06	-<br>16.69(7905)	<0.001	-	-0.13(-0.177,-0.083)	-0.406(-0.567,-0.216)
Worrier/anxious feelings	-<br>0.06	-<br>18.83(7905)	<0.001	-	-0.171(-0.217,-0.124)	-0.325(-0.5,-0.126)
**Happiness**	0.06	16.9(7891)	<0.001	0.063(0.056,0.07)	0.156(0.109,0.202)	0.348(0.151,0.519)

Table S2. Subjective well-being and FTSE100 scores: 5.5-year period. *The table shows stability of the market-mood relationships for the 5.5-year period (the same as the one investigated in the main analysis of brain-market relationships);* β_{std} *- standardized* β *coefficients,* p_{fdr} *– p-values corrected for multiple testing with false discovery rate. Subcomponents of negative emotions are binary variables (-), Day/MonthAVG – data averaged by days and months.*

Region	β_{std}	T_{776}	p_{fdr}
L Amygdala	-0.049	-6.24	<0.001
R Amygdala	-0.046	-5.76	<0.001
L Accumbens	-0.021	-2.92	0.008
R Accumbens	-0.025	-3.44	0.002
L LOFC	-0.022	-3.89	0.001
R LOFC	-0.009	-1.53	ns
L Insula	0.016	2.9	0.008
R Insula	0.02	3.47	0.002
L Subcallosal	0.013	2.29	0.042
R Subcallosal	0.011	1.82	ns
L Anterior Cingulate	-0.002	-0.26	ns
R Anterior Cingulate	0.005	0.78	ns
L Paracingulate	0.01	1.63	ns
R Paracingulate	0.011	1.78	ns

Table S3. ***Main results adjusted for intracranial volume, demographics, psychiatric diagnosis and seasonal effects.*** *The table shows stability of the identified relationships when controlling for age, sex, psychiatric diagnosis, seasonal effects (months) and intracranial volume (cerebrospinal fluid, white and grey matter); ns – non-significant.*

Region	Linear Mixed-Effects β_{std}	$T(df)$	p_{fdr}	Effect-sizes, Pearson r (95% C.I.) *Raw, n=30,775*	*DayAVG, n=1299*	*MonthAVG, n=66*
L Amygdala	-0.056	-4.2(878)	<0.001	-0.01(-0.021.0.002)	-0.052(-0.106.0.002)	-0.131(-0.362.0.114)
R Amygdala	-0.061	-4.96(878)	<0.001	-0.012(-0.023.-0.001)	-0.066(-0.12.-0.012)	-0.206(-0.427.0.038)
L Accumbens	-0.014	-1.1(878)	0.311	-0.001(-0.013.0.01)	-0.002(-0.057.0.052)	-0.029(-0.269.0.214)
R Accumbens	-0.01	-0.75(878)	0.488	-0.002(-0.013.0.009)	-0.004(-0.058.0.051)	-0.052(-0.29.0.192)
L LOFC	0.004	0.37(878)	0.713	<0.001(-0.011.0.011)	0.005(-0.049.0.059)	0.01(-0.233.0.251)
R LOFC	0.022	1.81(878)	0.088	0.003(-0.008.0.014)	0.037(-0.018.0.091)	0.101(-0.144.0.335)
L Insula	0.069	5.64(878)	<0.001	0.01(-0.001.0.021)	0.078(0.024.0.132)	0.197(-0.047.0.419)
R Insula	0.077	6.28(878)	<0.001	0.013(0.001.0.024)	0.089(0.034.0.142)	0.195(-0.049.0.417)
L Subcallosal	0.056	4.52(878)	<0.001	0.01(-0.001.0.021)	0.084(0.03.0.138)	0.203(-0.041.0.424)
R Subcallosal	0.048	3.88(878)	<0.001	0.009(-0.002.0.02)	0.083(0.029.0.137)	0.191(-0.053.0.414)
L Anterior Cingulate	0.044	3.79(878)	<0.001	0.009(-0.002.0.02)	0.071(0.017.0.125)	0.133(-0.112.0.364)
R Anterior Cingulate	0.047	3.93(878)	<0.001	0.009(-0.002.0.02)	0.066(0.012.0.12)	0.153(-0.092.0.381)
L Paracingulate	0.047	3.92(878)	<0.001	0.009(-0.002.0.021)	0.076(0.021.0.13)	0.211(-0.033.0.431)
R Paracingulate	0.041	3.4(878)	0.001	0.008(-0.003.0.019)	0.067(0.013.0.121)	0.176(-0.069.0.401)

Table S4. ***Main effects PCA-adjusted for the rest of the studied stock markets (first 5 principal components).*** *The table shows stability of the identified relationships when controlling for stock markets of the UK's 15 top trading partners [5], measured as first 5 principal components extracted from the merged time-series.*

	Interaction (linear term)			Main Effect		
	**β**	**T(df)**	p_{fdr}	**β**	**T(df)**	p_{fdr}
L Amygdala	0.01	1.24(776)	0.516	0.03	4.39(776)	<0.001
R Amygdala	<0.001	0.65(776)	0.887	0.01	1.9(776)	0.06
L Accumbens	<0.001	-0.81(776)	0.832	0.08	14.32(776)	<0.001
R Accumbens	<0.001	-0.16(776)	0.951	0.09	15.9(776)	<0.001
L LOFC	-0.01	-1.61(776)	0.429	0.06	12.35(776)	<0.001
R LOFC*	-0.01	-2.87(776)	0.05	0.06	11.92(776)	<0.001
L Insula	<0.001	-0.25(776)	0.951	0.02	4.32(776)	<0.001
R Insula	<0.001	0.4(776)	0.951	0.02	3.73(776)	<0.001
L Subcallosal	-0.01	-2.11(776)	0.211	0.02	4.39(776)	<0.001
R Subcallosal	-0.01	-1.28(776)	0.516	<0.001	-0.91(776)	0.363
L Anterior Cingulate	<0.001	0.33(776)	0.951	-0.03	-5.88(776)	<0.001
R Anterior Cingulate	<0.001	-0.06(776)	0.951	-0.03	-5.55(776)	<0.001
ICV	-0.01	-1.1(777)	0.273	0.15	28.52(777)	<0.001

Post-hoc correlation tests revealed the largest effect-sizes in lowest and highest-income citizens

Income (n)	***Pearson r (95% C.I.)***	***P-value***
Less than 18,000£ (4,557)	-0.045(-0.078,-0.012)	0.008
18,000-39,999£ (10,061)	-0.018(-0.04,0.005)	0.124
39,000-51,999£ (11,219)	-0.033(-0.055,-0.012)	0.002
52,000-100,000£ (8,570)	-0.027(-0.052,-0.002)	0.037
Greater than 100,000£ (2,699)	-0.067(-0.11,-0.024)	0.003

***Table S5**. Market-by-income interaction effect on the investigated volumetric brain measures, as well as the main of income.*

	Low Frequency		High Frequency	
	T_{881}	P_{uncor}	T_{881}	P_{uncor}
L Amygdala	-9.59	<0.001	-0.63	ns
R Amygdala	-11.71	<0.001	0.91	ns
L Accumbens	-9.78	<0.001	-0.44	ns
R Accumbens	-11.25	<0.001	0.33	ns
L LOFC	-5.06	<0.001	-2.02	0.043
R LOFC	-3.06	0.002	-2.12	0.034
L Insula	11.96	<0.001	-1.46	ns
R Insula	10.62	<0.001	-1.86	0.06
L Subcallosal	7.01	<0.001	-2.25	0.02
R Subcallosal	6.16	<0.001	-1.89	0.059
L Anterior Cingulate	6.27	<0.001	-0.19	ns
R Anterior Cingulate	6.23	<0.001	-0.44	ns
L Paracingulate	1.91	0.056	-0.93	ns
R Paracingulate	2.64	0.008	-2.17	0.034

Table S6. Brain-market associations: FTSE100 time-series deconvolved with Fast Fourier Transform (FFT) into low- and high-frequency bands. *Effects of low and high FTSE100 frequencies were estimated in one linear mixed-effects model assessing their independent contributions. The table shows that the main results are primarily driven by low-frequency oscillations, but high-frequency bands also exhibit some independent contribution.*

	LM: Brain~Market			LM: Market~Brain		
Lag	*AC*	*D-W*	*p*	*AC*	*D-W*	*p*
1	0.05	1.898	0.004	0.851	0.295	<0.001
2	0.002	1.994	0.82	0.842	0.313	<0.001
3	0.014	1.966	0.344	0.841	0.312	<0.001
4	0.017	1.958	0.296	0.84	0.314	<0.001
5	0.022	1.948	0.162	0.837	0.318	<0.001
6	0.029	1.932	0.062	0.837	0.318	<0.001
7	0.054	1.882	0.002	0.839	0.313	<0.001
8	0.095	1.8	<0.001	0.844	0.303	<0.001
9	0.004	1.981	0.636	0.829	0.334	<0.001
10	<0.001	1.989	0.908	0.827	0.337	<0.001

Table S7. Autocorrelations in the investigated time-series *The Durbin-Watson (D-W) test revealed significant autocorrelations (ACs) present in the day-averaged population brain and market data. The ACs were detected in both time-series but were particularly strong in the stock market data (Hurst exponent for the FTSE100 data was estimated at 0.87, suggesting presence of long-term positive autocorrelation).*

	H1: "Population Brain impacts Market"	***H2: "Market impacts Population Brain"***	***Portmanteau Stability Test***
L Amygdala	$\chi^2(df)=21.72(5), p=0.001$	$\chi^2(df)=26.28(5), p<0.001$	$L=5, \chi^2(df)=50.44(44), p=0.234$
R Amygdala	$\chi^2(df)=26.5(13), p=0.015$	$\chi^2(df)=35.76(13), p=0.001$	$L=13, \chi^2(df)=13.98(12), p=0.302$
L Accumbens	$\chi^2(df)=0.43(1), p=0.512$	$\chi^2(df)=2.49(1), p=0.115$	$L=1, \chi^2(df)=63.65(60), p=0.349$
R Accumbens	$\chi^2(df)=0.13(1), p=0.718$	$\chi^2(df)=5.75(1), p=0.016$	$L=1, \chi^2(df)=72.39(60), p=0.131$
L LOFC	$\chi^2(df)=0.5(1), p=0.481$	$\chi^2(df)=2.92(1), p=0.087$	$L=1, \chi^2(df)=60.35(60), p=0.463$
R LOFC	$\chi^2(df)=0(1), p=0.996$	$\chi^2(df)=0.04(1), p=0.841$	$L=1, \chi^2(df)=65.54(60), p=0.291$
L Insula	$\chi^2(df)=13.57(11), p=0.258$	$\chi^2(df)=16.52(11), p=0.123$	$L=11, \chi^2(df)=20.86(20), p=0.406$
R Insula	$\chi^2(df)=13.65(11), p=0.253$	$\chi^2(df)=15.38(11), p=0.166$	$L=11, \chi^2(df)=25.92(20), p=0.168$
L Subcallosal	$\chi^2(df)=18.82(8), p=0.016$	$\chi^2(df)=16.31(8), p=0.038$	$L=8, \chi^2(df)=36.65(32), p=0.262$
R Subcallosal	$\chi^2(df)=16.54(8), p=0.035$	$\chi^2(df)=19.44(8), p=0.013$	$L=8, \chi^2(df)=37.55(32), p=0.23$
L Anterior Cingulate	$\chi^2(df)=7.19(5), p=0.207$	$\chi^2(df)=20.44(5), p=0.001$	$L=5, \chi^2(df)=61.26(44), p=0.043$
R Anterior Cingulate	$\chi^2(df)=0.05(1), p=0.829$	$\chi^2(df)=21.41(1), p<0.001$	$L=1, \chi^2(df)=116.67(60), p<0.001$

Table S8. Causal relationships between the studied brain variables and market oscillations (daily scale). *For all the regions that passed the Portmanteau stability test for residual serial correlation (highlighted in bold), hypothesis 2 (H2: "Market impacts Polulation Brain") received slightly more support compared to hypothesis 1 (H2: "Population Brain impacts Market") with the optimal lag length (L) determined according to AIC criterion. However, the H2 could not be ruled out, as it was also equivalently supported for amygdala and subcallosal cortex.*

	H1: "Population Mood impacts Market"	***H2: "Market impacts Population Mood"***	***Portmanteau Stability Test***
NegEm	χ^2(df)=2.38(2), p=0.304	χ^2(df)=7.1(2), p=0.029	L=2, χ^2(df)=61.64(56), p=0.281
Irritability	χ^2(df)=3.21(3), p=0.36	χ^2(df)=9.59(3), p=0.022	L=3, χ^2(df)=63.43(52), p=0.133
Sensitivity/hurt feelings	χ^2(df)=1.36(1), p=0.243	χ^2(df)=10.73(1), p=0.001	L=1, χ^2(df)=69.56(60), p=0.187
Nervous feelings	χ^2(df)=4.03(2), p=0.133	χ^2(df)=0.16(2), p=0.925	L=2, χ^2(df)=79.12(56), p=0.023
Worrier/anxious feelings	χ^2(df)=0.06(1), p=0.803	χ^2(df)=10.4(1), p=0.001	L=1, χ^2(df)=86.02(60), p=0.015
Happiness	χ^2(df)=4.22(2), p=0.121	χ^2(df)=15.31(2), p<0.001	L=2, χ^2(df)=69.92(56), p=0.1

***Table S9**. **Causal relationships between the studied mood variables and market oscillations (daily scale, 14 years).**For all the regions that passed the Portmanteau stability test for residual serial correlation (highlighted in bold), hypothesis 2 (H2: "Market impacts Polulation Mood") received consistently more support compared to hypothesis 1 (H2: "Population Mood impacts Market") with the optimal lag length (L) determined according to AIC criterion. Measures that passed the Portmanteau stability test for residual serial correlation are highlighted in bold. L - optimal lag length determined according to AIC criterion.*

	Shuffled			Shifted			1/f Noise*			1/f Noise* (PCA-corrected*)		
	β_{std}	T	Pfdr	β_{std}	T	Pfdr	β_{std}	T	Pfdr	β_{std}	T	Pfdr
**L Amygdala**	0.002	0.38(810)	0.928	-0.015	-2.81(758)	0.006	-0.005	-0.93(758)	0.35	0.009	1.34(563)	0.261
**R Amygdala**	-0.004	-0.71(810)	0.919	-0.012	-2.28(758)	0.026	-0.007	-1.2(758)	0.266	0.003	0.45(563)	0.7
**L Accumbens**	-0.005	-0.89(810)	0.919	-0.052	-10.49(758)	<0.001	-0.021	-3.88(758)	<0.001	-0.004	-0.56(563)	0.666
**R Accumbens**	<0.001	0.09(810)	0.928	-0.053	-10.67(758)	<0.001	-0.026	-4.75(758)	<0.001	-0.009	-1.42(563)	0.261
**L LOFC**	-0.002	-0.51(810)	0.919	-0.028	-8.37(758)	<0.001	-0.018	-3.82(758)	<0.001	-0.007	-1.31(563)	0.261
**R LOFC**	<0.001	-0.1(810)	0.928	-0.036	-10.74(758)	<0.001	-0.023	-4.84(758)	<0.001	-0.008	-1.51(563)	0.249
**L Insula**	-0.002	-0.53(810)	0.919	-0.002	-0.49(758)	0.623	-0.008	-1.78(758)	0.113	-0.017	-2.9(563)	0.046
**R Insula**	-0.001	-0.15(810)	0.928	-0.006	-1.63(758)	0.111	-0.008	-1.68(758)	0.118	-0.014	-2.51(563)	0.046
**L Subcallosal**	-0.003	-0.54(810)	0.919	-0.022	-6.04(758)	<0.001	-0.016	-3.35(758)	0.002	-0.015	-2.61(563)	0.046
**R Subcallosal**	-0.003	-0.53(810)	0.919	-0.012	-3.3(758)	0.002	-0.01	-2.04(758)	0.069	-0.014	-2.39(563)	0.051
**L Anterior Cingulate**	0.003	0.74(810)	0.919	0.01	3.23(758)	0.002	0.004	1.05(758)	0.317	-0.004	-0.7(563)	0.602
**R Anterior Cingulate**	0.005	1.07(810)	0.919	0.009	2.81(758)	0.006	0.007	1.68(758)	0.118	-0.001	-0.25(563)	0.805
**L Paracingulate**	-0.001	-0.24(810)	0.928	-0.031	-9.81(758)	<0.001	-0.018	-4.13(758)	<0.001	-0.009	-1.7(563)	0.225
**R Paracingulate**	-0.002	-0.51(810)	0.919	-0.031	-9.61(758)	<0.001	-0.021	-4.72(758)	<0.001	-0.015	-2.75(563)	0.046
**Intracranial Volume**	-0.004	-1.16(810)	0.919	-0.033	-17.86(758)	<0.001	-0.024	-7.51(758)	<0.001	-0.006	-1.5(563)	0.249

Table S10. Effects of autocorrelations on the studied associations. *The table demonstrates effects of autocorrelations present in stock market time-series. The effects are not present in the shuffled data, but appear for the 1.5-years-shifted market time-series. The same effect (including directional relationships) can be induced with 1/f noise, but it disappears after adjusting the results for the rest of the studied non-UK markets (first 5 Principal Components).*
Note that when reporting effect-sizes of 1/f noise, we focused on a single simulation. Estimates from multiple simulations ultimately converge to zero (Supplement Fig. S12**) due to inconsistent directionality of the associations.*

	Raw	***DayAVG***	***MonthAVG***
Negative Emotions (total)	0.003/0.004	0.254/0.049	0.461/0.136
Irritability	-	0.172/0.059	0.23/0.178
Sensitivity/hurt feelings	-	0.262/0.081	0.522/0.255
Nervous feelings	-	0.216/0.078	0.415/0.066
Worrier/anxious feelings	-	0.224/0.047	0.459/0.197
Happiness	0.001/0.002	0.145/0.078	0.243/0.061
Diastolic blood pressure	0.007/0.014	0.23/0.049	0.363/0.134
Alcohol (intake frequency)	0.002/0.003	0.132/0.056	0.236/0.093
Alcohol (composite score intake)	0.008/0.015	0.167/0.064	0.36/0.058
L Amygdala	0.018	0.111	0.317
R Amygdala	0.018	0.119	0.314
L Accumbens	0.018	0.096	0.432
R Accumbens	0.019	0.106	0.556
L LOFC	0.017	0.085	0.191

R LOFC	0.016	0.067	0.173
L Insula	0.016	0.091	0.294
R Insula	0.016	0.078	0.209
L Subcallosal	0.015	0.083	0.138
R Subcallosal	0.014	0.072	0.138
L Anterior Cingulate	0.016	0.1	0.267
R Anterior Cingulate	0.016	0.091	0.256
L Paracingulate	0.016	0.066	0.051
R Paracingulate	0.017	0.066	0.143

Table S11. Associations between FTSE100 and the main investigated variables tested with mutual information criterion. *Two values per test are provided for the non-brain variables: 14 years (full dataset) / 5.5 years (MRI subsample). Subcomponents of negative emotions are binary variables (-), Day/MonthAVG – data averaged by days and months. Raw - individual measures without day-averaging.*

	UK (FTSE100)			China (SSEC)		
	**ARMA F | p-value**	**EXP F | p-value**	**GAUS F | p-value**	**ARMA F | p-value**	**EXP F | p-value**	**GAUS F | p-value**
**L Amygdala**	6.497| 0.0132	13.8762| <0.001	7.0036| 0.0103	2.3388| 0.0978	2.2776| 0.1079	2.3232| 0.1058
**R Amygdala**	0.0173| 0.8956	0.1885| 0.6657	10.14| 0.0022	2.5629| 0.1148	0.448| 0.5058	3.9355| 0.0521
**L Accumbens**	0.7944| 0.3762	0.9736| 0.3276	1.0369| 0.3125	1.477| 0.2289	1.2863| 0.2611	1.2851| 0.2613
**R Accumbens**	5.0228| 0.0285	3.5327| 0.0647	4.1783| 0.0451	0.8693| 0.3548	0| 0.9979	0.0035| 0.9529
**L LOFC**	1.8687| 0.1764	1.8823| 0.1749	1.8819| 0.1749	0.2356| 0.6291	0.4391| 0.51	0.4391| 0.51
**R LOFC**	9e-04| 0.9758	0.0561| 0.8135	0.0366| 0.8489	0.3142| 0.5771	0.2526| 0.617	0.2566| 0.6143
**L Insula**	2.8991| 0.0935	4.1395| 0.046	9.0609| 0.0037	0.332| 0.5665	0.263| 0.6098	0.9178| 0.2912
**R Insula**	1.4911| 0.2265	2.2419| 0.1392	6.5969| 0.0125	0.5494| 0.4613	0.4252| 0.5167	0.6595| 0.4198
**L Subcallosal**	2.0588| 0.1562	2.7478| 0.1023	6.4915| 0.0132	0.0328| 0.8568	0.0012| 0.9726	1e-04| 0.9923
**R Subcallosal**	0.3763| 0.5418	0.8152| 0.37	2.8113| 0.0985	NC	NC	NC
**L Anterior Cingulate**	2.1041| 0.1518	3.0764| 0.0842	5.1918| 0.026	1.6241| 0.2496	0.0949| 0.7591	2.9017| 0.0295
**R Anterior Cingulate**	1.5926| 0.2116	2.3858| 0.1274	4.3729| 0.0405	4.8736| 0.0041	0.0241| 0.8772	4.2607| 0.0058
**L Paracingulate**	15.6304| <0.001	9.0015| 0.0038	10.797| 0.0016	NC	NC	NC
**R Paracingulate**	5.0598| 0.0279	7.5846| 0.0076	10.8841| 0.0016	0.0039| 0.9505	0.0032| 0.9553	0.0234| 0.8789
**Intracranial Volume**	0.9185| 0.3415	1.8966| 0.1733	3.1058| 0.0828	0.16| 0.6905	0.0501| 0.8236	0.1019| 0.7506

Table S12. Replication of the main results employing Mixed Generalized Additive Modelling.

F | p-value – F-statistics and corresponding significance under various assumptions of autocorrelation structure: ARMA - autoregressive moving average process, with arbitrary orders for the autoregressive and moving average components, EXP - exponential spatial correlation, GAUS - Gaussian spatial correlation. NC – model did not converge.

	NegEm	Market	Unemployment	HP
**NegEm**	1	-0.56	0.46	-0.21
**Market**	-0.56	1	-0.75	0.56
**Unemployment**	0.46	-0.75	1	-0.27
**HP**	-0.21	0.56	-0.27	1

Table S13. Cross-correlations between mood and various socioeconomic metrics (monthly scale)

NegEm – Negative Emotions; Market – FTSE100;
HP – Housing Prices;
Unemployment – monthly unemployment rates (sourced from https://www.ons.gov.uk/economy/inflationandpriceindices/bulletins/housepriceindex/november).

	Housing Prices				Unemployment			
	Betas*	T	Pfdr	MonthAVG.n=99	Betas*	T	Pfdr	MonthAVG.n=66
L Amygdala	4.136	8.51(1426)	<0.001	0.412(0.188.0.594)	17.171	8.68(1426)	<0.001	0.366(0.136.0.558)
R Amygdala	5.621	10.39(1426)	<0.001	0.521(0.319.0.677)	24.242	11.07(1426)	<0.001	0.512(0.308.0.671)
L Accumbens	4.259	18.74(1426)	<0.001	0.792(0.68.0.868)	17.089	18(1426)	<0.001	0.766(0.643.0.85)
R Accumbens	4.222	19.97(1426)	<0.001	0.814(0.713.0.882)	17.39	19.83(1426)	<0.001	0.811(0.707.0.88)
L LOFC	17.621	14.47(1426)	<0.001	0.594(0.412.0.731)	76.774	12.95(1426)	<0.001	0.582(0.396.0.722)
R LOFC	17.458	15.73(1426)	<0.001	0.507(0.302.0.667)	71.392	13.28(1426)	<0.001	0.483(0.272.0.649)
L Insula	-0.851	-0.86(1426)	0.419	-0.262(-0.474.-0.021)	-12.37	-2.59(1426)	0.011	-0.2(-0.422.0.044)
R Insula	0.586	0.6(1426)	0.55	-0.183(-0.407.0.062)	-5.185	-1.09(1426)	0.293	-0.129(-0.36.0.116)
L Subcallosal	3.716	5.68(1426)	<0.001	-0.059(-0.297.0.185)	11.071	3.62(1426)	<0.001	-0.018(-0.259.0.225)
R Subcallosal	1.518	2.6(1426)	0.011	-0.129(-0.36.0.116)	2.561	0.94(1426)	0.347	-0.1(-0.334.0.146)
L Anterior Cingulate	-9.098	-6.49(1426)	<0.001	-0.256(-0.469.-0.015)	-45.801	-6.52(1426)	<0.001	-0.2(-0.421.0.044)
R Anterior Cingulate	-10.601	-6.62(1426)	<0.001	-0.313(-0.516.-0.077)	-54.14	-6.84(1426)	<0.001	-0.273(-0.483.-0.034)
L Paracingulate	16.133	13.92(1426)	<0.001	0.305(0.068.0.509)	67.765	11.65(1426)	<0.001	0.31(0.073.0.513)
R Paracingulate	16.143	12.98(1426)	<0.001	0.28(0.041.0.489)	63.751	10.59(1426)	<0.001	0.285(0.046.0.493)

*–non-standardized

Table S14. Structural characteristics of the studied fear and reward regions in relation to alternative socioeconomic indicators.

Overall, the analysis yielded results that were consistent with the main findings for FTSE100.

	Housing Prices				Unemployment			
	Betas*	T	Pfdr	MonthAVG.n=99	Betas*	T	Pfdr	MonthAVG.n=99
**NegEm (total)**	-0.002	-6.93(55484)	<0.001	-0.208(-0.368.-0.035)	0.034	21(55484)	<0.001	0.456(0.307.0.583)
Irritability	<0.001	-0.51(55484)	0.607	-0.149(-0.315.0.025)	0.003	5.71(55484)	<0.001	0.152(-0.022.0.317)
Sensitivity/hurt	-0.001	-10.86(55484)	<0.001	-0.232(-0.39.-0.061)	0.01	19.91(55484)	<0.001	0.382(0.223.0.521)
Nervous	<0.001	-0.58(55484)	0.607	-0.192(-0.354.-0.019)	0.003	7.55(55484)	<0.001	0.41(0.255.0.545)
Worrier/anxious	<0.001	-0.89(55484)	0.561	-0.048(-0.22.0.127)	0.004	7.76(55484)	<0.001	0.404(0.248.0.54)

**Happiness**	-0.001	-2.67(23307)	0.015	0.251(0.056.0.427)	-0.022	-23.75(23307)	<0.001	-0.469(-0.61.-0.299)

*–non-standardized

Table S15. Subjective well-being and alternative socioeconomic indicators

NegEm – negative emotions

The results were consistent for Unemployment rates. However, contrary to expectations, increases in housing prices were associated with more positive sentiment in the subjective wellbeing. Possibly, due the fact that in this specific sample increases in housing prices reflect economic growth, as also confirmed by positive correlation (r=0.56) of this metric with FTSE100 (see S13).

	Betas*	T	Pfdr
**NegEm**	<0.001	-22.24(36178)	<0.001
**Happiness**	<0.001	2.27(14817)	0.023
**L Amygdala**	-0.027	-6.49(881)	<0.001
**R Amygdala**	-0.032	-6.87(881)	<0.001
**L Accumbens**	-0.001	-0.67(881)	0.54
**R Accumbens**	-0.002	-1.14(881)	0.32
**L LOFC**	-0.003	-0.27(881)	0.791
**R LOFC**	0.012	0.99(881)	0.371
**L Insula**	0.061	5.83(881)	<0.001
**R Insula**	0.062	5.98(881)	<0.001
**L Subcallosal**	0.033	4.94(881)	<0.001
**R Subcallosal**	0.018	3.13(881)	0.003
**L Anterior Cingulate**	0.051	3.2(881)	0.002
**R Anterior Cingulate**	0.046	2.61(881)	0.012
**L Paracingulate**	0.064	4.96(881)	<0.001
**R Paracingulate**	0.066	5.07(881)	<0.001

*–non-standardized

Table S16. Main results adjusted for monthly UK housing prices and unemployment rate.

Overall, the analysis yielded consistent results. However, when adjusted for housing prices and unemployment rates, the effects of the stock market dynamics on subjective wellbeing became negligibly small. This was not the case for the primary outcomes (brain data).

References

1. World Medical Association Declaration of Helsinki: Ethical Principles for Medical Research Involving Human Subjects. *JAMA* **310**, 2191 (2013).
2. Toda, H. Y. & Yamamoto, T. Statistical inference in vector autoregressions with possibly integrated processes. *J. Econom.* **66**, 225–250 (1995).
3. Liu, M., Hu, X. & Schedl, M. The relation of culture, socio-economics, and friendship to music preferences: A large-scale, cross-country study. *PLOS ONE* **13**, e0208186 (2018).
4. Simonsohn, U., Simmons, J. P. & Nelson, L. D. Specification Curve: Descriptive and Inferential Statistics on All Reasonable Specifications. *SSRN Electron. J.* (2015) doi:10.2139/ssrn.2694998.
5. United Kingdom's 15 top import partners (Worldexports data). http://www.worldstopexports.com.
